# Computational Modeling and Self-Assembly Synthesis of Borazine-Based Free-Standing Molecular-Thin Films

**DOI:** 10.1021/acs.langmuir.5c05963

**Published:** 2026-01-05

**Authors:** Dario Calvani, Andy Jiao, Thomas J.F. Kock, Maxime A. Siegler, Karthick Babu Sai Sankar Gupta, Dmitri V. Filippov, Huub J. M. de Groot, G. J. Agur Sevink, Grégory F. Schneider, Francesco Buda

**Affiliations:** † Leiden Institute of Chemistry, Faculty of Science, 4496 Leiden University, 2333 CC Leiden, The Netherlands; ‡ 28414 Helmholtz-Zentrum Dresden-Rossendorf (HZDR), Bautzner Landstrasse 400, 01328 Dresden, Germany; § Center for Advanced Systems Understanding (CASUS), Conrad-Schiedt-Strasse 20, 02826 Görlitz, Germany; ∥ Department of Chemistry, 1466 Johns Hopkins University, Baltimore, Maryland 21218, United States

## Abstract

Boron-nitride-rich organic thin materials based on borazines have gained significant attention for their potential in nano­(opto)­electronic and energy storage devices. We address synthetic challenges in producing effective borazine-based thin films by proposing a dual theoretical and experimental protocol. This combines a multiscale computational approach, using density functional theory and classical molecular dynamics, with synthesis and thin-film formation via the Langmuir–Blodgett technique. The computational modeling focuses on three key properties: π–π stacking interactions, molecular steric hindrance, and dynamic self-assembly orientation. This modeling guided the selection of a borazine molecular building block and enabled the successful experimental formation of a free-standing molecular-thin borazine-based film. Solely π–π stacking interactions were found to drive the formation of a bilayer film with a molecular thickness of 2.1 nm, capable of spanning 0.6 μm diameter holes as a free-standing film. The agreement between theory and experiment confirms that the film retains essential features of the borazine molecular crystal, particularly intermolecular offset face-to-face π–π stacking and hexagonal-based pattern orientations. We thus establish a robust and transferable approach for modeling and synthesizing borazine-based thin materials, deepen the understanding of molecular interactions in borazine self-assembly, and demonstrate the suitability of the Langmuir–Blodgett technique for fabricating borazine-based 2D materials.

## Graphical abstract

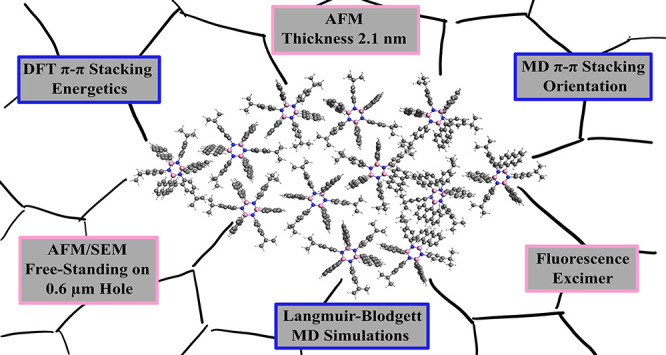

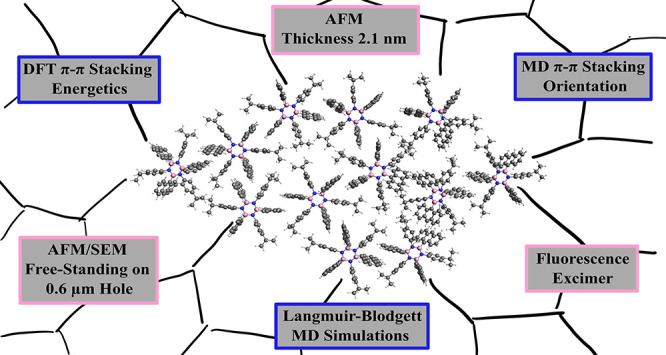

## Introduction

Over the past two decades, developments in borazine and boron-nitride-doped polycyclic aromatic hydrocarbon chemistry have spurred scientific interest in the theoretical design and experimental synthesis of novel hybrid molecular systems based on boron-carbon-nitrogen. The material science applications are diverse,^1,2^ including gas–liquid separation, water desalination, sensing techniques, nano­(opto)­electronics, and energy storage devices.^3,4^ The 2005 seminal work by Wakamiya and co-workers laid a cornerstone for research in borazine synthesis and their application in nano­(opto)­electronics by demonstrating the efficient synthesis of borazine-based aromatic bundle aggregates with a *C*_3_ symmetry and gear-shaped structures.^5^ They showed that the π–π stacking interactions between the aromatic moieties guide the aggregation and significantly influence the electronic behavior of the system.^5^ Successively, the Bonifazi group has developed, synthesized, and extensively characterized borazine-based molecular systems, yielding various crystal structures with diverse polymorphisms.^6–8^ They discussed the role of two-dimensional (2D) molecular-thick borazine-based materials for nano­(opto)­electronic applications,^6–8^ and recently also suggested a potential new direction toward borazine-based three-dimensional architectures.^9^ Bonifazi and co-workers extensively explored the 2D self-assembly of borazines on metal surfaces, providing a robust approach for studying the fundamental electronic interactions between the molecules and the metal surface, as well as their impact on borazine orientations. They demonstrated how specific aromatic peripheral groups control the orientation and strength of borazine π–π stacking self-assembly on metal surfaces via specific van der Waals and repulsive intermolecular forces.^10,11^ While self-assembly on a metal surface enables efficient planarization and interactions between the borazine building blocks, on the other hand, a strong interaction persists between the peripheral aromatic rings and the metal slab.^10,12^ This interaction likely results in extremely poor electronic decoupling of the aromatic and flexible carbon backbones from the metal surface,^13^ which irreversibly compromises the stability of borazine self-assembled materials during transfer to other substrates and subsequent manipulation. This instability ultimately causes permanent damage that limits the practical application of these materials.^2^

To overcome this key drawback and enable the effective application of borazine-based materials, the development of new, rational, and methodical approaches is essential. Molecular Langmuir–Blodgett bottom-up self-assembly at the water-to-air interface has shown great promise in producing 2D polycyclic aromatic hydrocarbon-based materials, showing an optimal integration of mechanical stability and chemical reproducibility.^14–16^ The selection of molecular building blocks and the careful balance of intermolecular interactions, such as π–π stacking and hydrogen bonding, between these molecular units and the water surface, are crucial for creating tailored 2D self-assembled materials with specific structural characteristics.^14–16^ Borazines are attractive candidates for Langmuir–Blodgett self-assembly at the water-to-air interface due to their ability to be functionalized with various sterically hindered aromatic groups, which can be translated into a variety of self-assembly morphologies through chemical versatility and tunability.^5,7,10,11^ Additionally, aromatic functionalized borazines exhibit mild reactivity in water, preventing undesired hydrolysis and expanding their application potential,^1,2^ which renders borazine Langmuir–Blodgett bottom-up self-assembly a timely research topic.

In this two-step study, we first perform an in silico analysis of π–π stacking motifs that drive self-assembly at the water-to-air interface for Wakamiya-type borazine molecular building block candidates.^5^ Second, we synthesize and characterize a borazine-based film with a promising motif using the Langmuir–Blodgett technique. The computational modeling focuses on Wakamiya-type borazines with a central *C*_3_ symmetric core functionalized with two alternating aromatic moieties (anthracene and phenyl-based groups) at each atom of the central borazine ring. In this work, four borazines, labeled in bold numbers **1**, **2**, **3**, and **4**, are considered; see Figure 1a–d. Of these, **2** and **3** have already been shown by Wakamiya et al. to be synthesizable with excellent yields and good stability under standard conditions.^5^ Borazine **1** is considered a proper alternative, though it has not yet been proven to be synthesizable. Borazine **4** is less sterically hindered, included only for comparison in the modeling study, also because its synthesis would likely be affected by hydrolysis. Moreover, borazines with anthracene groups connected to the nitrogens of the central borazine ring instead of the borons are not considered, as, to our knowledge, no synthesizable examples have been reported.^1,5,9^ We employ Density Functional Theory (DFT)-based methods in the first step to study the stacking interaction behavior and energetics of the borazines in a dimer configuration. Classical All-Atom Molecular Dynamics (MD) simulations are extensively employed to investigate interactions, orientations, and structure formation by self-assembly at the water-to-air interface under standard and Langmuir–Blodgett conditions. The simulations reveal that some borazines form a thin, π–π stacked, noncovalently bonded film with ordered patterns on the water surface. Particularly, the borazine core and aromatic edge moieties are identified to play a crucial role in determining the self-assembly structure through a balance of molecular symmetry, π–π stacking interactions, and steric hindrance.^1,7,10^ Properties such as π–π stacking energetics and molecular orientation are used to single out a promising borazine candidate from this small pool of borazines for synthesis and subsequent characterization, namely B,B′,B″-Tri­(9-anthryl)-*N*,*N*′,*N″*-tris­(p-isopropyl-phenyl) borazine (borazine **2**, Figure 1b). A molecular-thick borazine **2**-based film was experimentally produced via the Langmuir–Blodgett technique, transferred onto a silicon wafer, and characterized using atomic force microscopy (AFM), scanning electron microscopy (SEM), and fluorescence spectroscopy. This borazine **2**-based film demonstrated free-standing mechanical stability, with structural-mechanical properties, such as molecular thickness, strongly correlated with the theoretical predictions.

**Figure 1:**
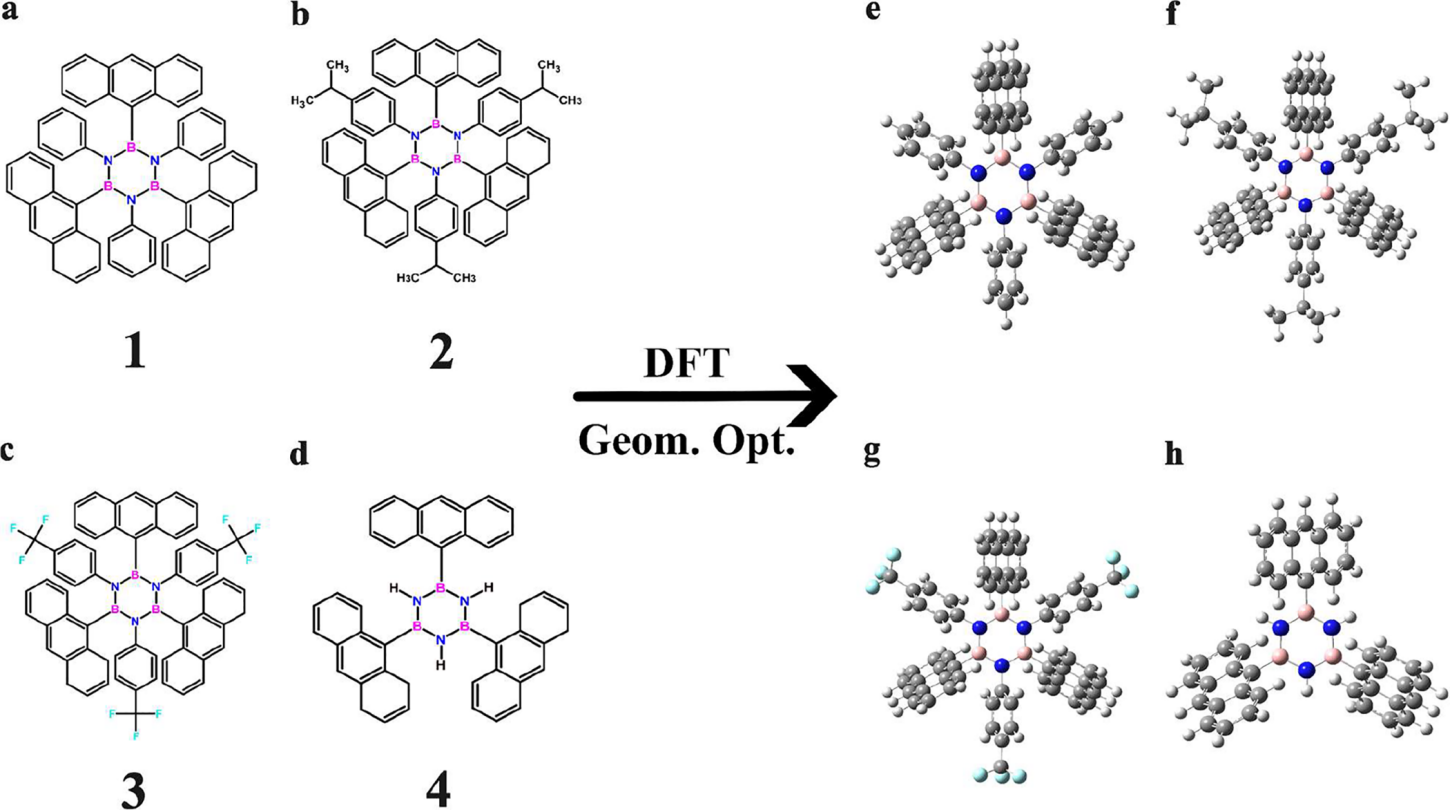
(a–d) Sketch structures of each borazine considered in the computational modeling of this work, **1**, **2**, **3**, and **4**, respectively. (e–h) Molecular ball and stick representation of the corresponding DFT optimized geometry of the monomers, with hydrogens in white, boron in pink, carbon in gray, nitrogen in blue, and fluorine in cyan, respectively.

This work represents a successful example of experimental synthesis of a π–π stacking-driven, bottom-up self-assembly of borazines via the Langmuir–Blodgett technique following in-silico modeling, yielding a stable, free-standing, boron-nitride-rich, molecular-thick organic film. The ultimate goal of this research is to establish an efficient, chemically precise method for the production and tuning of boron-nitride-rich carbon-based nanomaterials, with concrete applications ranging from nano­(opto)­electronics to energy and CO_2_ storage devices, as well as separation membranes, all contributing to a successful transition to sustainable energy.^1,2^

## Materials and Methods

### Computational Methods: DFT Calculations

Geometry optimization with frequency analysis of the borazine **1**, **2**, **3**, and **4** molecules (monomers) in the gas phase was performed using the DFT method with the PBE0 functional,^17,18^ D3­(BJ) correction,^19^ and 6-31G­(d,p) basis set, using the Gaussian 16 program suite.^20^ The maximum force is set to ≤1.5 × 10^–5^ Hartree Bohr^–1^ (RMS force ≤ 1.0 × 10^–5^ Hartree Bohr^–1^) and max displacement convergence criteria set to ≤6 × 10^–5^ Bohr (RMS displacement ≤ 4 × 10^–5^ Bohr). To assess the contributions of π–π stacking within the borazines, geometry optimizations were conducted for dimers of identical borazine **1**, **2**, **3**, and **4** molecules using the same level and approach of calculation employed for the monomers. The π–π stacking interaction for each dimer was estimated by subtracting twice the energy of the monomer from the total energy of the dimer, using the formula Δ*E* = *E*_dimer_ – 2 · *E*_monomer_, for borazine dimer **1**, **2**, **3**, and **4**, respectively. To compute the energy variation associated with changing the C–C–B–N dihedral angle in each borazine monomer, a series of DFT scans of one representative C–C–B–N dihedral angle was performed. These scans utilized the PBE0 functional,^17,18^ D3­(BJ) correction,^19^ and 6-31G­(d,p) basis set implemented through the Gaussian 16 program suite.^20^

To compute the NMR chemical shifts of the borazine **2**, single-point calculations on the optimized monomer and dimer structures were performed at the DFT level with the PBE functional,^21^ D3­(BJ) correction,^19^ and TZP basis set, using AMS software.^22^

### Computational Methods: All-Atom MD Simulations of the Water Slab

All MD simulations were carried out using the GROMACS 2021 software suite.^23–29^ The Particle Mesh Ewald method was employed to accurately account for electrostatic interactions.^30^ The cutoff for Coulomb and Lennard-Jones interactions was set to 10 Å. During the NVT simulation, the temperature was kept fixed with the V-rescale coupling method.^15,31^

The model for the water slab, comprised of 4139 water molecules, was simulated in a periodic box 5.0 × 5.0 × 20.0 nm^3^ using the TIP4P-Ew/2004 force field.^32^ The water surface tension was used as defined within the GROMACS software suite according to

$$\gamma_{\mathrm{water}}=\frac{1}{2}L_{z}\left[P_{zz}-\frac{1}{2}(P_{xx}+P_{yy})\right]$$

where *L*_*z*_ is the box length in the *z*-direction, *P*_*xx*_, *P*_*yy*_, and *P*_*zz*_ are the respective *xx*, *yy*, and *zz* elements of the pressure tensor,^33^ and the 12 originates from the presence of two *x–y* plane surfaces in the system. The system was first energetically minimized and then equilibrated for 8 ns with NVT at 300 K to obtain an average surface tension of γ_water_ = 58.46 mN. This value is in good agreement with previous studies.^34,35^ The water model was further validated using a radial distribution function and density analysis.^15^ Borazines **1**, **2**, **3**, and **4** were simulated using the All-Atom Optimized Potential for Liquid Simulations (OPLS-AA) force field,^36–38^ with parameters calculated via the LigParGen^39^ parametrization tools and for the central borazine ring obtained by reference boron-nitride OPLS-AA parameter.^40^ Partial charges were calculated with the CM5 model,^41^ and derived from single points calculations on the optimized borazine **1**, **2**, **3**, and **4** monomer structures performed at DFT level with the PBE0 functional,^17,18^ D3­(BJ) correction,^19^ and 6-31G­(d,p) basis set, using Gaussian 16 program suite.^20^ All MD results were illustrated using Visual Molecular Dynamics (VMD).^42^ The OPLS-AA parameters for borazine **1**, **2**, **3**, and **4** are reported in the Data Availability section.

### Computational Methods: All-Atom MD Simulations of the Borazines at Water–Air Interface and under Langmuir–Blodgett-like Conditions

Random input positions of 1, 2, 6, or 12 borazine molecules in a range of 10 Å above both water surfaces were generated using the PACKMOL18 program.^43^ Having two independent water-borazine interfaces provides a more symmetric MD simulation box and increases the statistics of the results by averaging over both interfaces compared to having only one water-to-borazine interface. Moreover, the presence of the borazines on both sides avoids the diffusion of the water molecule through the periodic boundary condition along the *z*-axis. The two interfaces can be considered independent due to the 5 nm thickness of the water box and a vacuum space of at least 5 nm above both the borazine molecules distributions along the *z*-axis (Figure S1). For each simulation, the system was first equilibrated with an NVT simulation at 70 K for 5 ns, and the temperature was then raised to 300 K for another 5 ns of NVT simulation. After these equilibrations, the production MD consisted of an NVT simulation run at 300 K for 10 ns. The final configuration extracted from the production MD was used as a starting point for the subsequent NPT surface tension simulations. For each borazine system, **1**, **2**, **3**, and **4** (Figure 1), we ran an independent series of MD simulations. Data presented in the manuscript were obtained by averaging the values on the final 10 ns of each corresponding production MD simulation.

For 12 molecules of borazine **2**, surface tension MD simulations were performed, using a surface tension coupling for surfaces parallel to the *x–y* plane. It employs normal pressure coupling for the *z*-direction. The surface pressure was then increased stepwise to generate the surface pressure *vs* MMA isotherm. At each chosen surface pressure (0, 0.25, 0.5, 1, 1.5, 2, 2.5, 3 mN m^–1^), the surface tension coupling MD has been performed while monitoring the change of the area in the *x*–*y* plane. We employed the Berendsen barostat in GROMACS for constant surface-tension simulations since it is the only barostat in GROMACS that supports surface-tension coupling.^25^ This choice is also supported by previous literature.^15^ For this coupling method to be effective, a value for the compressibility is required, which is close to the real compressibility of the system, namely 4.5 × 10^–5^ bar^–1^.^44^ Simulations were stopped after 10 ns, at which point an equilibrium state was reached. We ran three independent production MD simulations for each chosen surface pressure (0, 0.25, 0.5, 1, 1.5, 2, 2.5, 3 mN m^–1^). Data presented in the manuscript were obtained by averaging the values over the total 10 ns of each corresponding production MD simulation at each surface pressure. The slope for the theoretical isotherm at 2 mN m^–1^ was determined using linear regression on data points ranging from 1.5 and 2.5 mN m^–1^.

### Computational Methods: DFT and TD-DFT Simulations of Absorption and Fluorescence Spectra

The DFT and TD-DFT calculations for the absorption and fluorescence spectra on the borazine **2** were conducted using the CAM-B3LYP functional,^45^ D3­(BJ) correction^19^ and 6-31G­(d,p) basis set, following previous benchmarking on similar molecules.^46^ These calculations were performed employing the Gaussian 16 program suite.^20^ We computed the lowest three singlet excited states, which cover excitations up to approximately 3.54 eV, encompassing the full relevant UV–vis range observed experimentally. The ground state and excited state geometries, and the corresponding vibrational frequencies, have been determined at DFT and TD-DFT levels, respectively, including implicit continuum solvent effects for the CHCl_3_ with the PCM scheme.^47^ Starting from the calculated harmonic vibrational spectra of the ground state and excited state, the vibrationally resolved spectra were computed using the FClasses3 program.^48^ This computation employed a time-dependent (TD) approach that is eigenstate-free, relying on the fast evaluation of the analytical expressions for the time-correlation functions and their Fourier transform.^48^ The reported spectra have been simulated using convoluting Lorentzian functions presenting a half-width at half-maximum (HWHM) that has been adjusted to allow meaningful comparison with experiments (0.021 eV).^48^ FCclasses3 is one of the most effective codes for computing vibrationally resolved spectra; it uses the Franck–Condon approximation,^49,50^ and selects only the relevant vibronic contributions. It is mostly applied within the harmonic approximation for both initial and final states. In this case, an adiabatic (AH) scheme has been used,^51^ which expands the potential energy surface (PES) of the final state around its equilibrium geometry.

### Experimental Methods: Synthesis of Borazine 2

Borazine **2** was synthesized according to the literature,^5^ with minor alterations. 4-isopropyl aniline (0.190 mL, 1.41 mmol, 1 equiv) in dry, degassed toluene (2 mL) was added dropwise to a solution of BCl_3_ in heptane at 0 °C in a flame-dried Schlenk flask under a constant flow of N_2_. The solution was refluxed and stirred overnight. Purging and freeze–pump–thawing were not sufficient for the complete removal of the formed HCl. The mixture was then added to a solution containing an excess amount of 9-lithioanthracene at 0 °C, which was prepared from 9-bromoanthracene (1.60 g, 6.22 mmol, 4.4 equiv) and *t*-BuLi (7.3 mL, 12.4 mmol, 8.8 eq, 1.7 M in pentane) in THF (12 mL) at −78 °C under N_2_. The mixture was allowed to warm to RT and was stirred overnight. Then, water was added (5 mL), and an aqueous layer was extracted with CHCl_3_ (3 × 10 mL). The combined organic layer was dried with MgSO_4_, filtered, and evaporated under reduced pressure. The trianthryl-substituted borazine **2** was purified using silica column chromatography (toluene in pentane 40% → 100%). The slow diffusion of pentane into a solution of borazine **2** in toluene or DCM yielded the product as yellow crystals (78 mg, 0.081 mmol, 17%). See Section S3 in the Supporting Information for more details.

### Experimental Methods: Characterization of Borazine 2

The borazine **2** was characterized by NMR, mass spectrometry, elemental analysis, single crystal X-ray diffraction (SCXRD), and solid-state NMR (SS-NMR) (Supporting Information, Sections S3 and S4). Liquid NMR measurements were performed on a Bruker Avance-III-HD 850 MHz standard bore liquid-state NMR spectrometer with a 19.96 T magnetic field. In this field, ^13^C and ^1^H resonate at 213.84 and 850.33 MHz, respectively. A 5 mm cryoprobe (type CPTCI ^1^H–^13^C/^15^N/D) with a Z gradient system was used. The 90-degree pulses used for the proton and carbon experiments were 8.8 and 12 μs at 10 W and 135 W. A 5 mm NMR tubes (Z172600) were purchased from Cortecnet. Deuterated solvents were purchased from Eurisotop.

SS-NMR measurements were performed with a Bruker Neo console 750 MHz wide bore SS-NMR spectrometer in a 17.6 T magnetic field. In this field, ^13^C and ^1^H resonate at 188.66 and 750.23 MHz, respectively. A standard 3.2 mm triple resonance E-free magic angle spinning (MAS) probe was used. All the samples were packed in 3.2 mm thick-walled zirconium rotors with vessel caps and were spun around the magic angle (54.74) at spinning frequencies of 15 or 20 kHz. The temperature was kept constant at 298 K.

### Experimental Methods: Preparation and Deposition of Langmuir Films

Langmuir films were prepared with a KSV NIMA instrument equipped with a Teflon Langmuir trough (24,300 mm^2^), Delrin barriers, a platinum Wilhelmy plate, and a dipper, using ultrapure Milli-Q water as the subphase. Varying concentrations of **2** in CHCl_3_ were prepared by diluting an initial stock solution of 1 mg mL^–1^ with CHCl_3_ until the desired concentration. The spreading of the borazine **2** in CHCl_3_ solution was performed by carefully approaching the water surface with droplets hanging from the airtight glass syringes until physical contact. After 20 min, compression was started at a rate of 2 mm min^–1^ until the desired surface pressure was reached and maintained for 15 min. Langmuir films were transferred onto Si/SiO_2_ and quartz substrates via the Langmuir–Blodgett method by slowly pulling the substrate (typically 1 × 2 cm) upward vertically (0.05 mm min^–1^ unless specified otherwise) from the subphase at constant surface pressure. Langmuir films were transferred onto copper/QUANTIFOIL TEM grids via the Langmuir–Schaefer method. See also Supporting Information, Sections S5 and S6, for more details. The slope for the experimental isotherm at 2 mN m^–1^ was determined using linear regression on data points ranging from 1.5 and 2.5 mN m^–1^.

### Experimental Methods: Characterization of Borazine 2-Based Films

Atomic force microscopy (AFM) was performed using a PK Nanowizard 4 Ultra Speed AFM. Height analysis was performed in AC mode on Langmuir–Blodgett films deposited on Si/SiO_2_ wafers using tips with a resonance frequency of 300 kHz and a spring constant of 26 mN m^–1^. Samples on Cu/QUANTIFOIL TEM grids were measured using tips with a resonant frequency of 70 kHz and a spring constant of 2 mN m^–1^. Scanning electron microscopy (SEM) was measured on an Apreo SEM instrument equipped with a CCD camera. Typical imaging conditions were a T2 detector with the Optiplan Use case, an acceleration voltage of 2 kV, and a beam current of 13–100 pA. Transmission electron microscopy (TEM) experiments were conducted on a Talos L120C microscope operated at 120 kV. UV–vis spectroscopy was performed on a Cary 60 (Agilent) instrument. Fluorescence spectroscopy was performed with an FLS900 fluorescence spectrometer equipped with a 450 W xenon lamp. Solutions were measured in a quartz cuvette cell with a path length of 1 cm. Langmuir–Blodgett and spin-coated films were measured on a quartz slide.

## Results and Discussion

### DFT Computational Modeling

For each of the four borazines considered (see Figure 1), initial DFT analysis via geometry optimization was performed for the monomers and respective dimers to estimate the π–π stacking interaction energies that could guide molecular self-assembly (see Computational Methods for the details). In Wakamiya-like borazines, the major contribution to π–π stacking interactions arises from the anthracene moieties.^5^ The anthracene–anthracene interaction is known to be stronger than the benzene–benzene interaction when normalized by the number of carbon atoms.^52^ Consequently, the optimized monomers were initially arranged such that one anthracene moiety of one monomer faces that of the other monomer, placed at a distance of 3.5 Å, within the typical range of anthracene-anthracene π–π stacking interaction, with an AB stacking configuration.^5^ The optimized geometries of the dimers reveal their preferred type of π-π stacking interaction for each borazine pair, along with their respective Kohn–Sham (KS) HOMO distributions, as depicted in Figure 2. The KS HOMOs of the monomers are shown in the Supporting Information, Section S2, Figure S2. All the monomer and dimer optimized structures were found to have no imaginary frequencies, confirming that they correspond to minima on their potential energy surfaces. The estimation and comparison of the π–π stacking interaction energy between the four Wakamiya-like borazines is presented in Figure 2, and detailed in the Computational Methods section. Systems **1** and **2** exhibit an offset face-to-face (OFF) or parallel-displaced π-π stacking,^53^ with average minimum C–C distances (*D*_min_(C_anth_ – C_anth_)), between the two anthracene groups of approximately 3.41 and 3.34 Å, respectively, as shown in Figure 2a,b, and schematically represented in Figure 2e. On the other hand, the distances between the centers of the aromatic rings in one anthracene moiety and the closest centers of the aromatic rings in the other anthracene moiety (*D*_center_(C_anth_ – C_anth_)) are approximately 3.95 and 3.80 Å, respectively, as reported in Figure 2a,b, and schematically represented in Figure 2e. Interestingly, borazine **2** displays a slightly firmer localization of the HOMO between the p-isopropyl-phenyl groups and the adjacent interacting anthracenes compared to borazine **1**. This can be attributed to the electron-donating effect of the p-isopropyl groups on the phenyl center, which increases the π electron cloud interacting with adjacent anthracenes, and reduces the C–C minimum distance between the interacting anthracene moieties by a dual π–π interacting and repulsive steric effect.^5^ This results in a stronger π-π stacking energy estimate for borazine **2** (−29.83 kcal mol^–1^) than borazine **1** (−19.19 kcal mol^–1^), as reported in Figure 2a,b. For the borazine **3** dimer, the withdrawing effect of the trifluoromethyl group (−CF_3_) present in the *para* position on each phenyl group, along with F–F and F−π interactions, drives the π–π stacking between anthracenes, preferably adopting an edge-to-face (EF) or T-shaped configuration,^53^ with average minimum distance between the two anthracene groups *D*_min_ ≈ 3.67 Å (*D*_center_ ≈ 4.55 Å), see Figure 2c.^54–56^ The π–π stacking energy estimate for borazine **3** amounts to −25.18 kcal mol^–1^, which is lower compared to the value for borazine **2**. Borazine **4** adopts a configuration that optimizes π-π stacking between the anthracenes with an average minimum distance of *D*_min_ ≈ 3.56 Å (*D*_center_ ≈ 4.86 Å), see Figure 2d. In this case, we noticed increased intercalation of the monomers, resulting from a reduced steric hindrance due to the absence of the phenyl moieties, and an enhanced rotation of the anthracene group relative to the borazine center along one of their equivalent carbon–carbon–boron–nitrogen (C–C–B–N) dihedrals compared to the borazines **1**, **2**, and **3** (see Supporting Information Figure S3 inset). Steric hindrance between the anthracene moieties in borazines **1**, **2**, and **3** plays a significant role in providing molecular rigidity and in orienting the π–π stacking in OFF and EF configurations within the dimer.^5,10^ To analyze the energetics of this torsional degree of freedom, DFT scan calculations along a specific C–C–B–N dihedral were performed for each of the four borazine systems (see Supporting Information Figure S3 inset). The variation of torsional energy of one representative C–C–B–N dihedral angle over the range of 0–180° is reported in Figure S3. Systems **1**, **2**, and **3** feature a maximum of around 60 kcal mol^–1^ at approximately 140° when the anthracene is nearly flat relative to the borazine center. In contrast, system **4** exhibits a lower energy barrier of approximately 20 kcal mol^–1^ at 120° for the C–C–B–N torsion, as it lacks the three bulky phenyl-based groups connected to the borazine’s nitrogen atoms of systems **1**, **2**, and **3**. This analysis provides insight into the variation in flexibility along the C–C–B–N dihedral angle for each borazine dimer. For the borazine **4** dimer, the higher flexibility and lower steric hindrance, compared to the other borazines, lead to a pronounced intercalated interaction between monomers. The π–π stacking energy estimate of −34.11 kcal mol^–1^ is higher than that of borazine **2**. This flexibility, the lower steric hindrance, and the π-π stacking energetics could promote disoriented π–π stacking interactions in larger poly-borazines systems. The DFT calculations provide a first clear rationale for selecting borazines based on dimeric packing and flexibility. However, to accurately explore and predict the borazine self-assembly orientation at the water-to-air interface on a nanometer structural level, All-Atom MD simulations are required.

**Figure 2:**
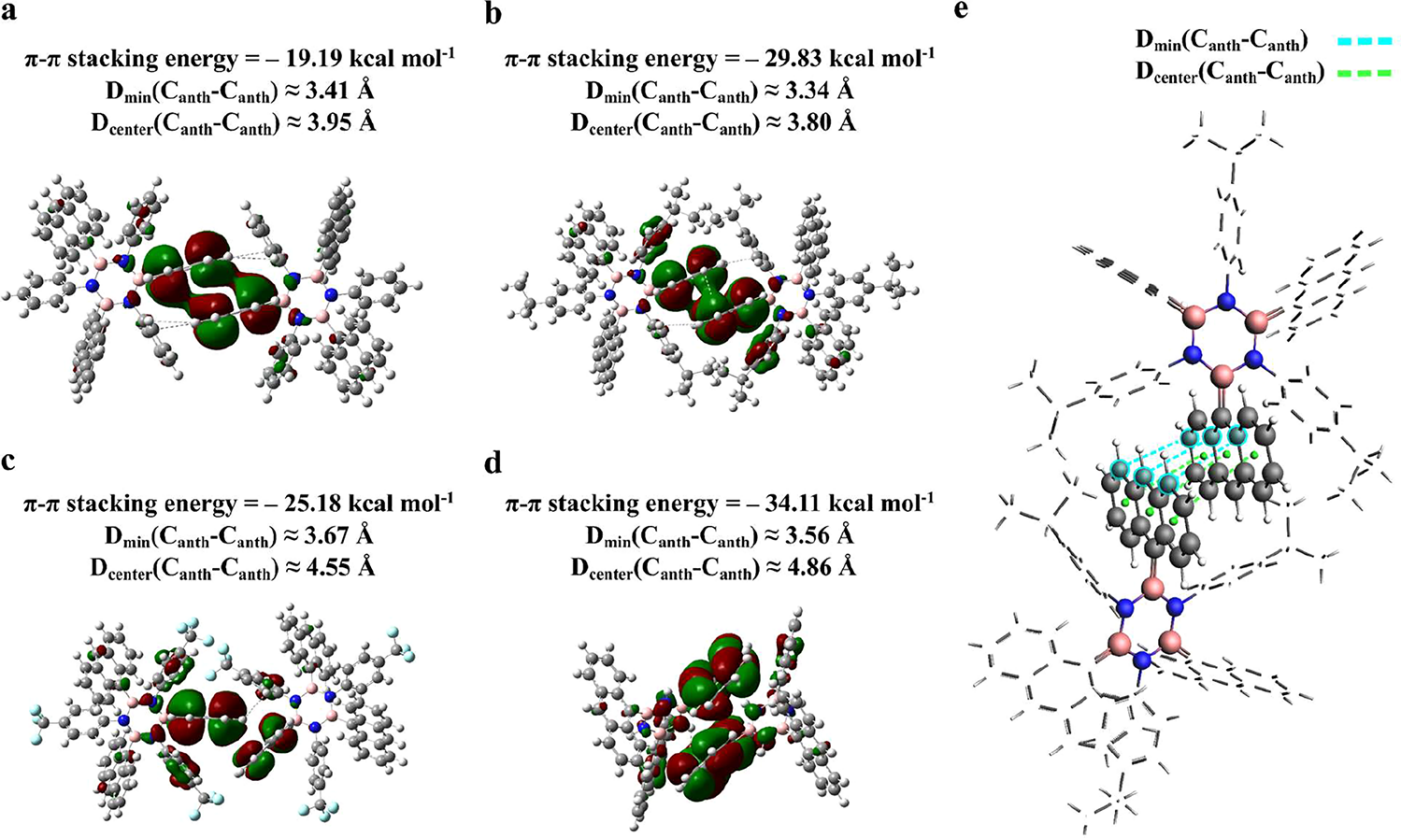
(a–d) DFT geometry optimized dimer configurations and pictorial representations of the KS HOMO for each type of borazine, 1, 2, 3, and 4, respectively, with isosurface value = 0.01. The π–π stacking interaction energy estimates (kcal mol^–1^), Δ*E* = *E*_dimer_ – 2 · *E*_monomer_, for borazine **1**, **2**, **3**, and **4**, dimers, respectively. (e) Schematic representation of the borazine 2 dimer illustrates the minimum carbon–carbon distance between two adjacent anthracene (anth) of two different monomers reported as *D*_min_(C_anth_ – C_anth_) with dashed cyan lines; the center carbon–carbon distance calculated as the distance between the center of each aromatic ring belonging to one anthracene moiety to the closest center of the aromatic rings of the other anthracene moiety is reported as *D*_center_(C_anth_ – C_anth_) with dashed green lines. In ball and stick, the molecular structures are shown, with hydrogens in white, boron in pink, carbon in gray, nitrogen in blue, and fluorine in cyan.

### All-Atom MD Computational Modeling

We performed All-Atom MD simulations to study the supra-molecular organization of borazines at the water-to-air interface and room temperature. A water slab was modeled to include two independent water–air interfaces, and various quantities of each borazine (1, 2, 6, or 12 molecules) were considered at the water surfaces (see Computational Methods for details). For borazines **1**, **2**, and **3**, MD results indicate that the molecules are arranged at the water surface with the central borazine core essentially parallel to the surface and with the aromatic edge moieties perpendicular to the surface (Figure 3a–c). In contrast, the self-assembly of borazines **4** gives rise to gross disorder (Figure 3d). Analysis of the tilt angle (*Φ*), as defined in Figure S4, indicates that as the number of **1**, **2**, or **3** molecules increases, the central borazine ring adopts an increasingly parallel configuration with respect to the water surface, and with tilt angle distributions in the range of 5°< *Φ* < 20°, see Figure 3e–g. Augmenting the number of borazines from 1 to 12 enhances the contribution of the π–π stacking interactions, which cooperatively dominate over thermally induced disorder, resulting in a hexagonal-based pattern at the water-to-air interface (see Figure 3a–c).^14,52^ This honeycomb-like orientation of borazines **1**, **2**, and **3** is governed by characteristic OFF and EF π–π stacking interactions between the aromatic groups as well as the *C*_3_ space group symmetry, producing a hexagonal gear-shaped motif in line with the seminal findings of Wakamiya et al.^5^ While the distribution of the tilt angle *Φ* for borazine **2** is consistently centered around 10° for varying numbers of molecules, a slight shift to 20° is observed for borazine **1** and **3**. In the case of borazine **1**, this shift can be related to the weaker π–π stacking interaction compared to borazine **2** and **3**, according to the DFT results. For borazine **3**, on the other hand, this shift can be explained by the temporary formation of hydrogen bonds between the fluorine atoms in the trifluoromethyl groups and the oxygens of the water molecules at the surface. Hydrogen bonding can compete with the π-π stacking interactions and tends to reorient the borazines **3**.^14^ The interactions between the nonamphiphilic borazines **1**, **2**, and **3** and the water slab are primarily repulsive, which likely results in structures that are more decoupled from the water surface compared to those observed on metal surfaces.^10^ In the borazine **4** system, the lack of aromatic substituents on the borazine’s nitrogen atoms leads to weak steric hindrance. This allows for energetically stable intercalated but disoriented self-assembly configurations above the water surface (Figure 3d), characterized by a broad tilt angle distribution ranging from 10° to 90°, in stark contrast to borazines **1**, **2**, and **3** (Figure 3h). The nonmonotonic trend of the tilt angle in borazine **4** exhibits varying magnitudes depending on the number of molecules. Each MD trajectory used for the analysis was found to be equilibrated (Figure S5). We therefore attribute this behavior of the borazine **4** case to weak steric hindrance between the anthracene moieties of each molecule, leading to high disorder in the orientation with respect to the water surface. The self-assembly disorder observed for borazine **4** is also attributed to a more favorable rotation of the anthracene moieties compared to the other borazines, detected in the DFT analysis of the C–C–B–N torsion. Accordingly, based on the favorable π–π stacking interactions (Figure 2b), the optimal orientation on the water surface at room temperature (Figure 3f), and the good synthetic yield previously reported by Wakamiya and co-workers,^4^ borazine **2** was selected for synthesis and consequent bottom-up film self-assembly via the Langmuir–Blodgett technique.

**Figure 3:**
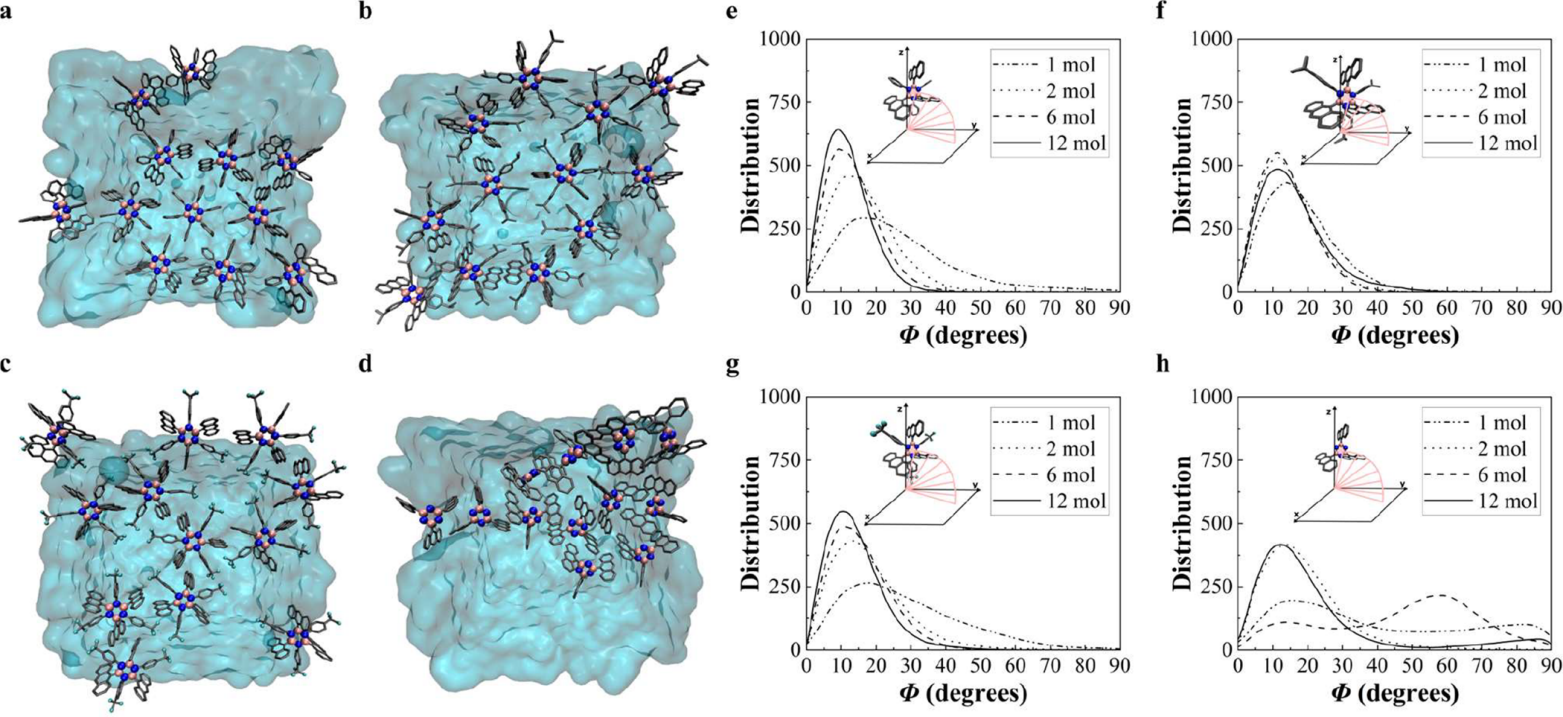
Representative MD snapshots of 12 molecules at the interface with the water surface after the MD equilibration at 300 K for each borazine homologue: (a–d) correspond to the borazine systems **1**, **2**, **3**, and **4**, respectively. The borazines are represented by balls and sticks: boron, carbon, nitrogen, and fluorine are colored in pink, gray, blue, and cyan, respectively; hydrogens are omitted for clarity. The water slab is represented as a light-blue surface. Distribution of the tilt angle (*Φ*) (e–h) per each borazine system **1**, **2**, **3**, and **4**, respectively, as derived from the MD simulations with 1, 2, 6, and 12 molecules (mol). The definition of the tilt angle *Φ* as the arc between the plane of the borazine center of each borazine (pink) and the *x–y* plane of the water surface is illustrated in the insets. The borazines are represented by balls and sticks: boron, carbon, nitrogen, and fluorine are colored in pink, gray, blue, and cyan, respectively; hydrogens are omitted for clarity. Distribution curves were obtained via Gaussian broadening with default standard deviation and normalized per amount of borazine molecules, using a kernel density estimation to produce these plots.

### Synthesis and Characterization of Borazine 2

The borazine **2** was synthesized according to the Wakamiya and co-workers’ procedure,^5^ with slight modifications. The borazine core was prepared by (1 + 1′ + 1 + 1′ + 1 + 1′) hexamerization of isopropyl aniline and BCl_3_. The borazine core was reacted with an excess amount of 9-lithioanthracene to avoid quenching by the formed HCl byproduct. Borazine **2** was obtained with a 17% yield and characterized using liquid NMR, mass spectrometry, elemental analysis, SCXRD, and SS-NMR (Supporting Information, Sections S3 and S4, and Figures S6–S15). We crystallized borazine **2** by slow diffusion of pentane into a solution of borazine **2** in DCM:toluene 1:1 (v/v). In terms of crystal packing characterization, the SCXRD analysis (Tables S1 and S2) revealed π–π stacking distances where the anthracene moieties of different borazines **2** are π–π stacked offset face-to-face (OFF) with an average center anthracene-anthracene π–π distance (*D*_center_(C_anth_ – C_anth_)) of 3.9–4.8 Å (Figure S11). The orientation of the interacting borazines **2** and the π–π stacking distances match the configuration, and the average minimum and center anthracene-anthracene π–π distances of *D*_min_(C_anth_ – C_anth_) ≈ 3.6 Å and *D*_center_(C_anth_ – C_anth_) ≈ 4.6 Å, respectively, predicted by the MD simulations for the borazine **2**-based film at the water-to-air interface (Figure 3b). Larger π–π stacking distances are found in the SCXRD and MD simulations than in the DFT results. This can be explained by the increased cooperative π–π stacking interactions in an aromatic aggregate of multiple borazines **2** than in the dimer.^14^ Moreover, the borazine molecule orientations found in the SCXRD (Figure S11) and MD simulation (Figure 3b) are similar, with the rigid and nonamphiphilic behavior of the aromatic moieties showing the same hexagonal-based pattern due to stable and oriented OFF π–π stacking interactions among the aromatic groups. Interestingly, when single crystals of borazine **2** were slowly cooled from 203 K down to 110 K in 35–45 min, the distances between the anthracene moieties decreased with a crystal color shift from yellow to white, showing thermochromic properties at low temperatures (Figure S11).

To obtain more insights into the π–π stacking interactions between borazine **2** molecules within the crystal, we also performed SS-NMR via MAS NMR of the borazine **2** crystals (Supporting Information, Section S4, and Figures S12–S15). The chemical shifts of the solid-state borazine **2** sample are comparable to those for the molecule in solution, indicating a modest π–π interaction between borazine **2** molecules in the solid state as well as in solution (Table S3). The chemical shifts for the molecules in solution and in the solid state are listed in Table S3, which are also in line with the DFT theoretical predictions. The standard deviations for the NMR ^13^C signals between theory and experiments are ≈2.44 and ≈1.82 ppm for the DFT monomer *vs* liquid NMR (in DCM), and DFT dimer *vs* SS-NMR, respectively, demonstrating excellent agreement for the assignment of the NMR signals (Table S3). The ^1^H–^13^C heteronuclear dipolar correlation MAS NMR experiments provided the ^1^H resonance assignment of the aromatic moieties of the borazine **2**, in particular the isopropyl-phenyl, allowing probing of the ring currents related to the OFF π–π stacking in the crystal (Figure S15).^57^ The CH_2_ and CH_3_ signals at negative ppm values are evidence of cooperative aggregation in aromatic OFF π–π stacking packing.^57^ The borazine **2** crystal MAS NMR shows upfield aggregation shifts for the CH_2_ and CH_3_ isopropyl signals (Figure S15), confirming the presence of cooperative π–π stacking interactions in OFF packing. The splitting of the CH_2_ and CH_3_ isopropyl MAS NMR signals is further confirmed by the SCXRD analysis at 203 K, which reveals that two out of the three isopropyl groups are disordered. The carbons of these two isopropyl groups exist in two different orientations with specific occupancy factors of 0.70 vs 0.30 (for carbons C27/C28/C29), and 0.47 vs 0.53 (for carbons C37/C38/C39), respectively, as extracted from the crystallographic data (Table S1). Overall, this is in line with the OFF packing found in the DFT and MD simulations.

### Langmuir–Blodgett Film Fabrication, Surface-Tension Results, and Langmuir–Blodgett-like MD Simulations

It has previously been reported that the solute concentration of nonamphiphilic molecules can strongly affect the film morphology.^58–60^ To investigate the behavior of nonamphiphilic borazine **2**, different stock solutions of borazine **2** were prepared in chloroform, ranging from 0.5 to 0.05 mg mL^–1^. These solutions were then carefully spread at the water-to-air interface at room temperature, and the chloroform was allowed to evaporate (Figure 4a). The total amount of deposited borazine **2** on the water-to-air interface remained the same (23 nmol), while the total amount of chloroform changed from 44 to 440 μL to accommodate the different stock concentrations. After the evaporation of the chloroform, we initiated the compression of the Langmuir film in the Langmuir–Blodgett trough (Figure 4b), during which the surface pressure in mN m^–1^ and mean molecular area (MMA) in Å^2^ molecule^–1^ (Å^2^ molec^–1^) were monitored to record the compression isotherms (Figures 4c, S16, and S17a). Depending on the concentration of the starting stock solution, the compression produced different isotherms. The change in concentration does not affect the isotherm of amphiphilic molecules such as 1-palmitoyl-2-oleoyl-*sn*-glycero-3-phosphocholine (POPC) (Figure S17b) taken as an amphiphilic standard, suggesting that the shift in the isotherm for the borazine **2** system is due to the nonamphiphilic characteristics of borazine. We hypothesized that the difference in isotherms is caused by aggregation during the deposition step on water (Figure 4a); it has been reported that the spreading of similar anthracene molecules at the water-to-air interface displayed immediate crystal formation upon evaporation of the solvent.^16^ In our experiments, while the total amount of deposited borazine **2** remains the same, the amount of borazine **2** per droplet (and the number of droplets) depends on the concentration of the stock solution. More concentrated solutions result in more concentrated amounts of borazine **2** per droplet on the surface, which, due to its nonamphiphilic property, tends to aggregate into multilayer islands before the molecule could spread and equilibrate at the surface.^16^ Therefore, by lowering the concentration, we avoid the tendency of the spread borazine **2** to aggregate into possible multilayer islands. This can be observed by the shift of the isotherm toward higher MMA values at lower concentrations (Figure 4c), allowing the formation of increasingly homogeneous films on the surface of water. The tangent line drawn at 2 mN m^–1^ on the experimental green isotherm (Figure 4c) indicates the MMA corresponding to the film morphology after the compression is released during the transfer onto the silicon wafer, Si/SiO_2_, (Figure 4b).^61^

**Figure 4:**
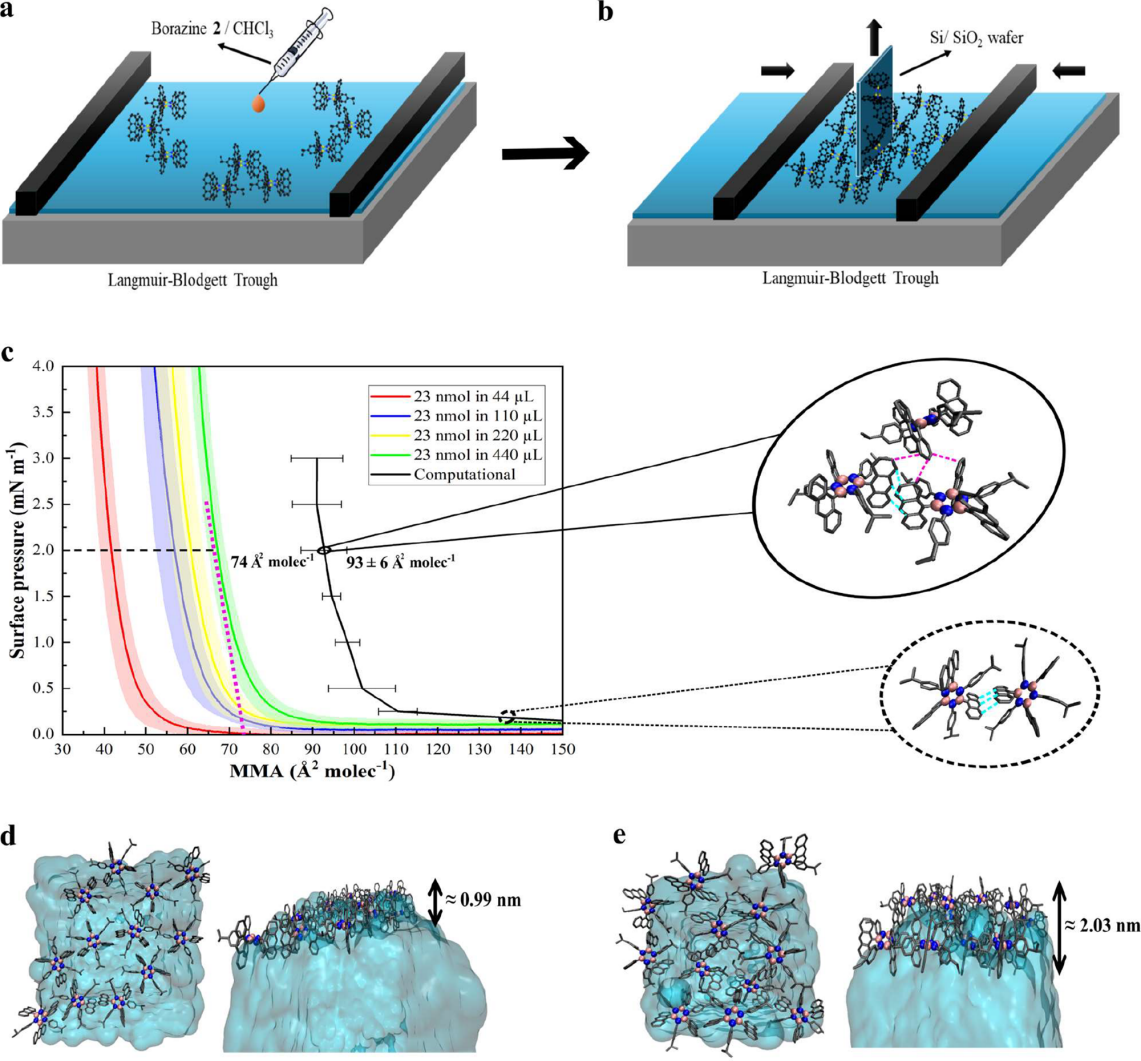
(a) Illustration of a Langmuir–Blodgett trough. A range of 0.5–0.05 mg mL^–1^ solutions of borazine **2** in chloroform were deposited dropwise at the water-to-air interface. (b) Illustration of a Langmuir–Blodgett trough after compressing the borazine 2 molecules above the water surface, and subsequent transfer by the Langmuir–Blodgett method of the thin film onto a silicon wafer (Si/SiO_2_) brought into contact with the compressed borazine **2** molecules. (c) Langmuir–Blodgett isotherms of the borazine **2** thin film after depositing 23 nmol borazine **2** in increasing amounts of CHCl_3_ (from 44 to 440 μL), where the surface pressure in mN m^–1^ is plotted *vs* the mean molecular area (MMA) in Å^2^ molec^–1^. The lighter-colored areas indicate the standard deviation for each corresponding isotherm. Upon dilution, the isotherm is shifted to higher MMA. The intersection of the extrapolated violet dashed line with the point of tangency with the green isotherm (23 nmol in 440 μL) at 2 mN m^–1^ indicates the MMA at which corresponds the morphology of a homogeneously distributed film. The solid black line is the isotherm computed via MD simulations under Langmuir–Blodgett-like conditions for a system of 12 borazine **2**. The MMA is averaged over three separate MD simulations with error bars indicating standard deviations. MD representative snapshots without surface pressure (bottom dashed black circle) and with surface pressure 2 mN m^–1^ (top solid black circle), and respective anthracene-anthracene π–π minimum distances *D*_min_(C_anth_ – C_anth_) within the bottom layer (cyan dashed line) and between bottom and top layers (violet dashed line). Top and side views of (d), the 12 borazines **2** system after MD equilibration at 300 K, and (e), the 12 borazines **2** system after MD compression at 2 mN m^–1^ surface pressure and 300 K, respectively. The borazine **2** molecules packing has a hexagonal-based pattern structure above the water surface involving all the anthracene moieties stacked in an offset face-to-face (OFF) configuration, consistent with the crystal structure reported in Figure S11. The thickness (black bifrontal arrow) for each system of 12 borazines **2** fluctuates between 0.99 nm for the system without surface pressure and around 2.03 nm for the Langmuir–Blodgett-like system at 2 mN m^–1^ surface pressure, indicating monolayer and bilayer assemblies, respectively. The borazine **2** is represented by balls and sticks: boron, carbon, nitrogen, and fluorine are colored in pink, gray, blue, and cyan, respectively, and hydrogens are omitted for clarity.

The surface pressure variation in the Langmuir–Blodgett experimental setup can be simulated via a computational approach of applying pressure in the *x–y* plane of the simulation box.^15^ Constant surface pressure simulations for six pressure values were performed for 12 borazines **2** on the water slab and repeated for three different starting conditions to gain information about statistics. Plotting surface pressure *vs* MMA yields an isotherm that enables direct comparison to the experimental data (Figure 4c). This shows the same trend between the computed and experimental isotherms, apart from a constant MMA offset (Figure 4c). Comparing MMAs at a surface pressure of 2 mN m^–1^ provides an estimate of about 19 Å^2^ for this offset. This value can be explained by the presence of a uniform distribution of borazine **2** monomers in one monolayer above the water slab at the beginning of the compression simulations (see Figure 4d), while most likely the experimental distribution of borazine **2** above the water surface before compression is heterogeneous. In particular, experimentally, initial aggregation can lead to the formation of sporadic cluster islands, which will decrease the MMA compared to the MMA for dispersed molecules. This interpretation is further supported by the finding that the slopes at a surface pressure of 2 mN m^–1^ for the experimental and computational isotherms are comparable, −0.30 mN molec m^–1^ Å^–1^ and −0.28 mN molec m^–1^ Å^–1^, respectively, suggesting the π–π stacking and packings are similar to each other and, consequently, mechanical properties such as elasticity.^61^ Focusing on the MD simulations, the thickness of the borazine **2**-based film before compression (without surface pressure) is approximately 1 nm and corresponds to a monolayer of borazine **2** molecules (Figure 4d). With the gradual increase of the surface pressure, the average thickness doubled with respect to the case at no surface pressure, as shown in Figure S18 by the density peaks of the borazine **2** molecules within the simulation box. For a surface pressure of 2 mN m^–1^, the average thickness increases to approximately 2 nm, which corresponds to the formation of a bilayer of borazine **2** molecules (see Figure 4e). This is further confirmed by the peak in the density of the borazine **2**-based film that doubled in width in the 2 mN m^–1^ case with respect to the no surface pressure one (Figure S18). In the case of a surface pressure of 2 mN m^–1^, the OFF π–π stacking between the anthracene moieties is retained (Figure 4c, circle snapshots) within the bottom layer. Specifically, in the MD simulation at a surface pressure of 2 mN m^–1^, the anthracene moieties exhibit characteristic OFF π–π stacking arrangements, with approximate average minimum and center anthracene-anthracene π–π distances of *D*_min_(C_anth_ – C_anth_) ≈ 3.5 Å and *D*_center_(C_anth_ – C_anth_) ≈ 4.8 Å, respectively, within the bottom layer. We highlight the presence of interactions between borazine **2** among the bottom and top layers (see Figure 4c solid circle snapshot, and Figure 4e). This interaction shows a typical OFF π–π stacking fashion between the lower part of the anthracene moieties of the top layer and the upper part of the anthracene moieties of the bottom layer (see Figure 4c solid circle snapshot and Figure 4e). The approximate average minimum and center anthracene-anthracene π–π distances among layers are *D*_min_(C_anth_ – C_anth_) ≈ 3.5 Å and D_center_(C_anth_ – C_anth_) ≈ 5.6 Å, respectively. As a consequence of the applied surface pressure, we observed a partial reorientation of the borazine **2** molecules above the water surface (Figure 4e), indicated by the slight shift in the tilt angle from ≈10–15° for the uncompressed system to ≈20–25° for the one at a surface pressure of 2 mN m^–1^(Figure S19). This rearrangement under compression is attributed to the OFF π–π stacking interactions among the anthracene moieties of the bottom and top borazine **2** layers (Figure 4c–e). The overall stability of the organized pattern under the Langmuir–Blodgett-like condition is attributed to the persistent OFF π–π stacking acting as a cooperative-oriented driving force between anthracene groups, outlining an entropy-harnessing effect along with the intrinsic rigidity of borazine **2**.^62–64^

### Langmuir–Blodgett Film Thickness Analysis via AFM and Freestanding Ability via SEM

The borazine thin films were formed by drop casting diluted solutions of borazine **2** in CHCl_3_ and subsequently compressing them in a Langmuir–Blodgett trough at different surface pressures (Figures 4c and S17a). The floating films were transferred onto the Si/SiO_2_ substrate using the Langmuir–Blodgett technique. Large uniform films with spanning areas of over 100 × 100 μm^2^ were found for the films obtained at a surface pressure of 2 mN m^–1^ (Figure 4c green line, Figures 5a, S20, and S21a). AFM was used to determine the average thickness by analyzing the height profile over a crack in the film (Figure 5a,b). These uniform, thin films show a thickness of 2.1 nm, corresponding to a bilayer of borazines **2** (Figure 5a,b). This value aligns well with the thickness extracted from the MD simulations for borazine **2** at 2 mN m^–1^ (Figure 4e). Then, we compared the Langmuir–Blodgett films obtained from 0.05 mg mL^–1^ at two different surface pressures: 2 *vs* 15 mN m^–1^. Although the flakes obtained at 15 mN m^–1^ surface pressure showed a thickness of 2.2 nm, they exhibited high degrees of aggregation (Figure S22a–c). This can be attributed to the presence of inhomogeneous clusters, characterized by elevated height levels observed in the AFM (Figure S22a–c). The borazine **2** molecules form a uniform bilayer at a low surface pressure of 2 mN m^–1^, and eventually collapse into multilayered films with inhomogeneous aggregates upon further compression at 15 mN m^–1^. To avoid aggregate formation, all production transfers were performed at 2 mN m^–1^. Afterward, we compared the quality of the films obtained from two different stock concentrations: 0.05 *vs* 0.2 mg mL^–1^. By changing the stock concentration from 0.05 to 0.2 mg mL^–1^, flakes spanning areas of 20 × 20 μm^2^ could be transferred with a similar thickness of 1.8 nm (Figure S22d–f). However, larger particles could be observed using the 0.2 mg mL^–1^ solution, characterized by bright spots in the AFM, resulting in the production of borazine **2**-based film with a smaller area due to a less stable morphology of the film compared to the solution with 0.05 mg mL^–1^. Although the thickness remains similar to the 0.05 mg mL^–1^ case, the overall quality of the Langmuir–Blodgett films derived from the higher concentration stock at 0.2 mg mL^–1^ appears reduced. Finally, to investigate whether the transfer speed on the Si/SiO_2_ substrate affects the resulting films, we analyzed films obtained from 0.05 mg mL^–1^ at a fast transfer speed of 2.5 mm min^–1^ (Figure S22g–i), compared to 0.5 mm min^–1^ (Figure 5a,b). At the faster transfer speed, the films were thinner and contained holes and clusters characterized by the bright spot in the AFM (Figure S22g–i). We investigated the thickness by analyzing the cross-section of the edge of the flake, which revealed a value of approximately 1 nm. This thickness corresponds to a monolayer borazine **2**-based film. The fast transfer produced flakes smaller than the 100 × 100 μm^2^ observed at the slower transfer speed of 0.5 mm min^–1^ (Figures 5a and S21a). Therefore, we hypothesize that a stable and uniform Langmuir–Blodgett borazine **2**-based film forms as a bilayer at a 2 mN m^–1^ surface pressure, 0.05 mg mL^–1^ stock concentration, and transfer speed onto the Si/SiO_2_ substrate of 0.5 mm min^–1^.

**Figure 5:**
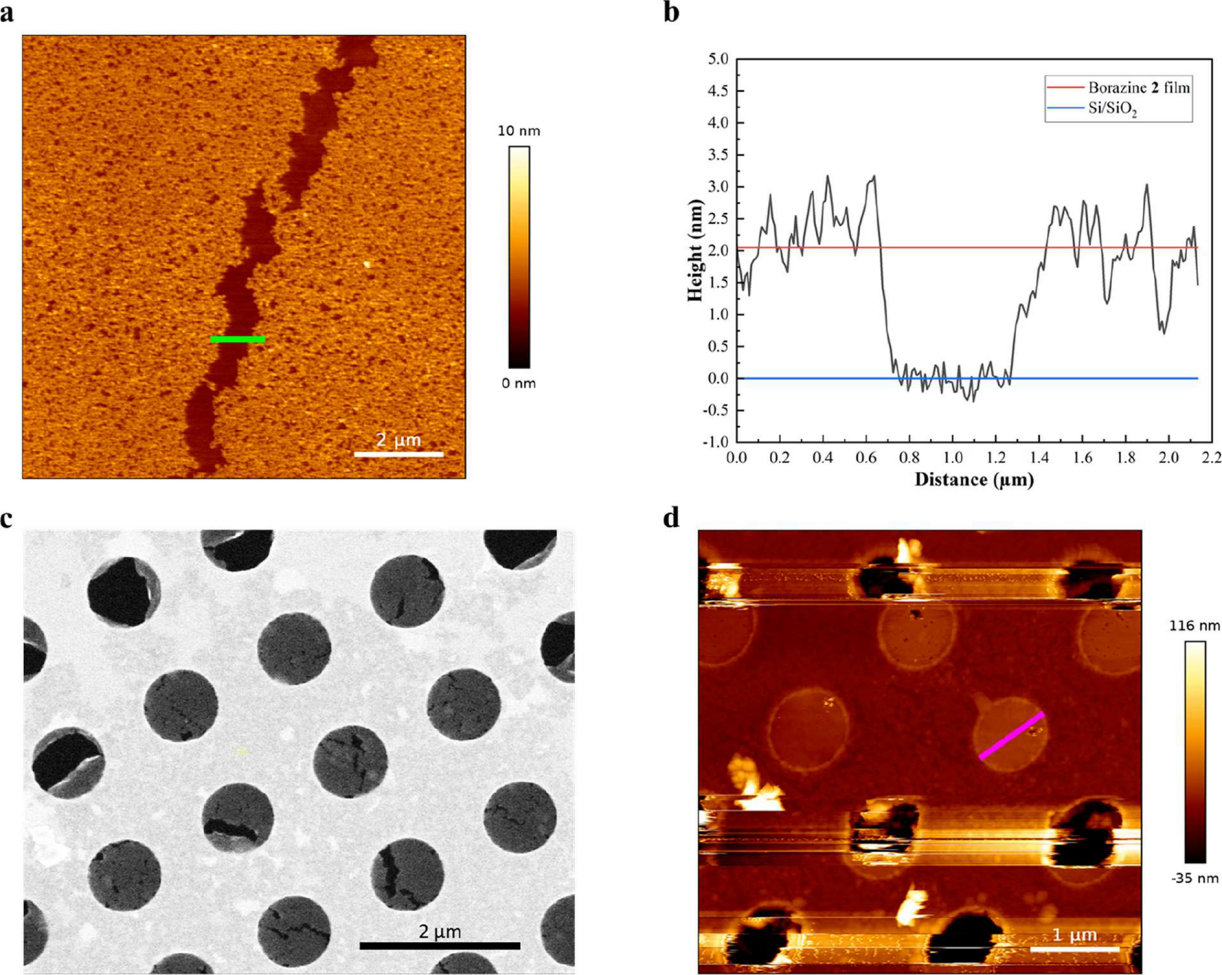
(a) AFM micrograph (10 μm × 10 μm) of a crack (green bar) in the borazine **2** film. (b) Height profile over the green cross-section, resulting in an estimated height of 2.1 nm calculated by the height difference between the average of the borazine (red line) and the Si/SiO_2_ heights (blue line). (c) SEM image collected at 25000× magnification (black scale bar, 2 μm) showing the Langmuir–Schaefer films prepared from a 0.05 mg mL^–1^ solution of borazine **2**, compressed to 2 mN m^–1^, and transfered on a QUANTIFOIL carbon film perforated with an array of 0.6 μm diameter holes on a copper TEM grid. (d) AFM images of a free-standing film obtained from a 0.05 mg mL^–1^ solution of borazine **2**, compressed to 2 mN m^–1^, and transfered by Langmuir–Schaefer on a QUANTIFOIL grid containing 0.6 μm diameter holes (magenta bar) in a copper TEM grid. The magnification of the AFM analysis is reported in Figure S21d.

To investigate the freestanding ability of the borazine **2**-based film, the Langmuir–Blodgett films created from 0.05 mg mL^–1^ borazine **2** in CHCl_3_ and compressed to 2 mN m^–1^ were transferred via the Langmuir–Schaefer technique onto copper TEM grids covered with a QUANTIFOIL layer containing 0.6 μm apertures. These borazine **2**-based films were found to be freestanding over 0.6 μm diameter holes (Figure 5c). However, when these films were exposed to the electron beam in the scanning electron microscope, holes appeared and grew larger in the films during the measurements (Figure S21b). We could not detect long-range crystallinity of the Langmuir borazine **2**-based film by selective area electron diffraction (SAED) (Figure S21c), presumably due to electron beam damage. To confirm that these holes are only formed during measurement and not during sample fabrication, AFM tapping mode was used on transferred samples before SEM measurements. Intact and stable freestanding films could be found over 0.6 μm holes (Figures 5d and S21d). In conclusion, consistent with the previous study on decacyclene-based Langmuir–Blodgett films,^15^ these results demonstrate that the intermolecular cooperative π-π stacking interactions between the aromatic moieties in the borazines **2**, anthracenes, and isopropyl-phenyls, are sufficiently strong to render the borazine **2**-based bilayer film freestanding over 0.6 μm diameter holes.

### Molecular Orientation via Fluorescence Spectroscopy

To confirm the packing mode of the borazine **2** molecules retained in the Langmuir–Blodgett films, UV–vis absorption and fluorescence spectroscopies were employed to measure absorption and fluorescence on the borazine **2**-based films transferred onto quartz. Compared to borazine **2** in the solution of CHCl_3_, a slightly red-shifted but similar absorption spectrum was observed for the Langmuir–Blodgett borazine **2**-based film (Figure 6a). This slight redshift in the absorption spectrum is an indication of overlapping anthracene moieties.^16,65,66^ On the other hand, fluorescence experiments on the Langmuir–Blodgett borazine **2**-based film revealed a large red-shifted broad signal, which is attributed to anthracene excimer formation in the solid state (Figure 6b). Excimer emission is typically broad, featureless, and significantly red-shifted relative to the well-defined vibronic emission of free, noninteracting anthracene units.^67^ The measurements also showed a characteristic broad and unstructured excimer emission centered around 500 nm, with no detectable vibronic features associated with isolated anthracenes. The occurrence of excimer emission is a reliable indication of interaction among anthracene moieties, reinforcing the idea of having the OFF (offset face-to-face) π–π stacking.^16,65,66,68^ In addition, the wavelength of solid-state anthracene excimer signals correlates with the overlap ratio between the two anthracene monomers, with an excimer peak around 489 nm for the borazine **2**-based film corresponding to an overlap of approximately 30%.^68^ Interestingly, the redshift in absorption and excimer spectra was also observed in the borazine **2**-based layer prepared by spin-coating from a solution of borazine **2** in CHCl_3_ on quartz (Figure 6a,b), indicating that borazine **2** rapidly self-assembles into an OFF π–π stacking motif. Consequently, the incorporation of poly­(methyl methacrylate) (PMMA) to physically separate the borazine **2** during spin-coating leads to the absence of the excimer emission (Figure 6b). Moreover, the UV–vis absorption and fluorescence spectra in CHCl_3_ were further analyzed via time-dependent DFT (TD-DFT) calculations in the polarizable continuum model (PCM) for CHCl_3_ solvation, and vibrationally resolved spectra were computed using the FCclasses3 program (Figure S23), showing a remarkable confirmation of the experimental data in CHCl_3_ solution.^48^ In summary, UV–vis measurements on the borazine **2**-based film, consistent with DFT calculations, MD simulations at the water-to-air interface, and in agreement with SCXRD and SS-NMR data, confirm the strong π–π stacking tendency of borazine **2** and its likely adoption of an OFF (offset face-to-face) stacking motif in both the crystal and the film. This corresponds to a borazine **2** hexagonal gear-shaped conformation in the crystal, which is expected to translate to a hexagonal-based pattern in the film.

**Figure 6:**
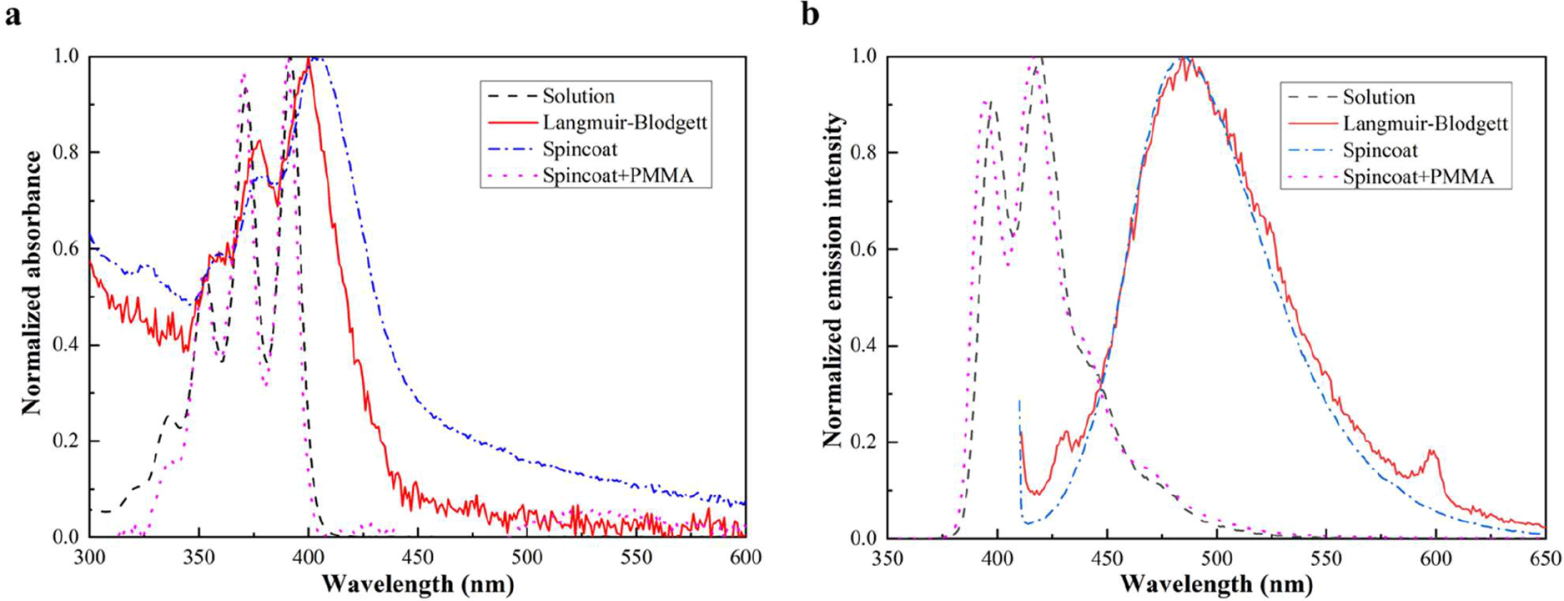
(a) Normalized absorption, and (b) fluorescence spectra: in black dash line, borazine **2** in solution (50 μM in CHCl_3_); in red solid line, borazine **2** film transferred by Langmuir–Blodgett on a quartz slide prepared from a 0.05 mg mL^–1^ borazine **2** solution in CHCl_3_, compressed to 2 mN m^–1^ and transferred with a speed of 0.5 mm min^–1^; in blue dash-dotted line, a spin-coated layer of borazine **2** (1 mg mL^–1^ in CHCl_3_, 80 μL, 500 rpm, 1 min); in magenta dotted line, a spin-coated layer of borazine **2** (1 mg mL^–1^ in CHCl_3_) diluted (1:1 v/v) with PMMA (600 kDa, 0.4 μM in anisole) (80 μL, 500 rpm, 2 min).

## Conclusions

We developed and described a combined computational–experimental approach to model and realize a molecularly thin free-standing film of a borazine-based compound assembled via the Langmuir–Blodgett method. Using a stepwise multiscale computational approach of DFT and classical All-Atom MD simulations, borazine **2** was identified as the most promising candidate among four different borazines due to its favorable π–π stacking energetics and stable hexagonal packing at the water-to-air interface. Experimental validation confirmed the successful synthesis and Langmuir–Blodgett assembly of borazine **2** into uniform bilayer films, with AFM-measured thickness (≈2.1 nm) in agreement with MD predictions. Fluorescence spectroscopy revealed excimer emission consistent with offset face-to-face π–π stacking, as also observed in the crystal structure by SCXRD and SS-NMR. These findings confirm that the self-assembly motif is preserved from crystal to film and is driven by directional π–π interactions. Despite minor limitations in crystallinity and electron beam stability, the films exhibited sufficient cohesion to span 0.6 μm apertures, confirming their freestanding nature. This work provides a proof of concept for the modeling and fabrication of 2D boron–nitrogen-rich organic nanomaterials based on borazine molecules driven solely by π–π stacking interactions. Future works could explore rational design starting from our fundamental model and applying it systematically to a wide borazine chemical space, including functionalized amphiphilic derivatives to enhance ordering and stability,^14^ as well as experimental covalent locking strategies such as photopolymerization.^16^ Overall, this study lays the groundwork for tunable, π–π stacked thin-film materials with potential applications in tunable nano­(opto)­electronics technologies such as organic light-emitting diodes (OLEDs), and energy storage devices.

## Supplementary Material



## Data Availability

The computational metadata with DFT-based and MD-based data, and the plots’ raw data are provided as supporting material openly available in ZENODO EU Open Research Repository at 10.5281/zenodo.15302655. CCDC 2409119 (2_110 K) and 2409120 (2_203 K) contain the supplementary crystallographic data for this paper. These data can be obtained free of charge from The Cambridge Crystallographic Data Centre via www.ccdc.cam.ac.uk/data_request/cif. All the rest of the data will be available upon a reasonable request.

## Notes

Additional references;^69^ additional computational and experimental analysis; computational methods; computational results; synthesis of borazine **2**; characterization of borazine **2**; Langmuir–Blodgett film fabrication, Langmuir–Blodgett-like MD simulations, and surface-tension results; Langmuir–Blodgett film thickness analysis via AFM and freestanding ability via SEM; fluorescence spectroscopy and TD-DFT calculations; and computational methods (DOCX)
