# Supplementary material for: Computational Modeling and Self-Assembly Synthesis of Borazine-Based Free-Standing Molecular-Thin Films
Source: Langmuir. 2026 Jan 5;42(2):2314–27. doi: 10.1021/acs.langmuir.5c05963 (PMC12825379; doi:10.1021/acs.langmuir.5c05963)
Supplement: Supplementary file 1 [file la5c05963_si_001.docx]

**SUPPORTING INFORMATION**

**Computational Modeling and Self-Assembly Synthesis of Borazine-Based Free-Standing Molecular-Thin Films**

*Dario Calvani*^*,†,‡,§^ *Andy Jiao,*^†^ *Thomas J.F. Kock,*^†^ *Maxime A. Siegler,*^‖^ *Karthick Babu Sai Sankar Gupta,*^†^ *Dmitri V. Filippov,*^†^ *Huub J. M. de Groot,*^†^ *G. J. Agur Sevink,*^†^ *Grégory F. Schneider,^*,^*^†^ *Francesco Buda.^*,^*^†^

^†^ Leiden Institute of Chemistry, Faculty of Science, Leiden University, 2333 CC Leiden, The Netherlands.

^‡^ Helmholtz-Zentrum Dresden-Rossendorf (HZDR), Bautzner Landstrasse 400, 01328 Dresden, Germany.

^§^ Center for Advanced Systems Understanding (CASUS), Conrad-Schiedt-Strasse 20, 02826 Görlitz, Germany.

^‖^ Department of Chemistry, Johns Hopkins University, MD 21218 Baltimore, USA.

* To whom correspondence should be addressed: [d.calvani@hzdr.de](mailto:d.calvani@hzdr.de), [g.f.schneider@chem.leidenuniv.nl](mailto:g.f.schneider@chem.leidenuniv.nl), [f.buda@lic.leidenuniv.nl](mailto:f.buda@lic.leidenuniv.nl)

**Number of pages:** 24

**Number of figures:** 23

**Number of tables:** 3

**S1. Computational Methods;**

**S2****.** **Computational Results;**

**S3.** **Synthesis of Borazine 2;**

**S4.** **Characterization of Borazine 2;**

**S5****. Langmuir-Blodgett Film Fabrication, Langmuir-Blodgett-like MD Simulations, and Surface-Tension Results;**

**S6.** **Langmuir-Blodgett Film Thickness Analysis via AFM and Freestanding Ability via SEM;**

**S7.** **Fluorescence Spectroscopy and TD-DFT Calculations;**

**References.**

**S1. Computational Methods**


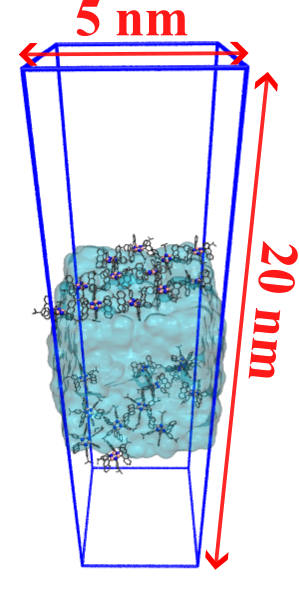


**Figure S1**. Schematic representation of the simulation box (5.0 × 5.0 × 20.0 nm^3^) used for all the simulations with borazine **2** represented by balls and sticks: boron, carbon, nitrogen, and fluorine are colored in pink, grey, blue, and cyan, respectively, hydrogens are omitted for clarity, and the water bulk shown with a light-blue surface.

**S2. Computational Results**


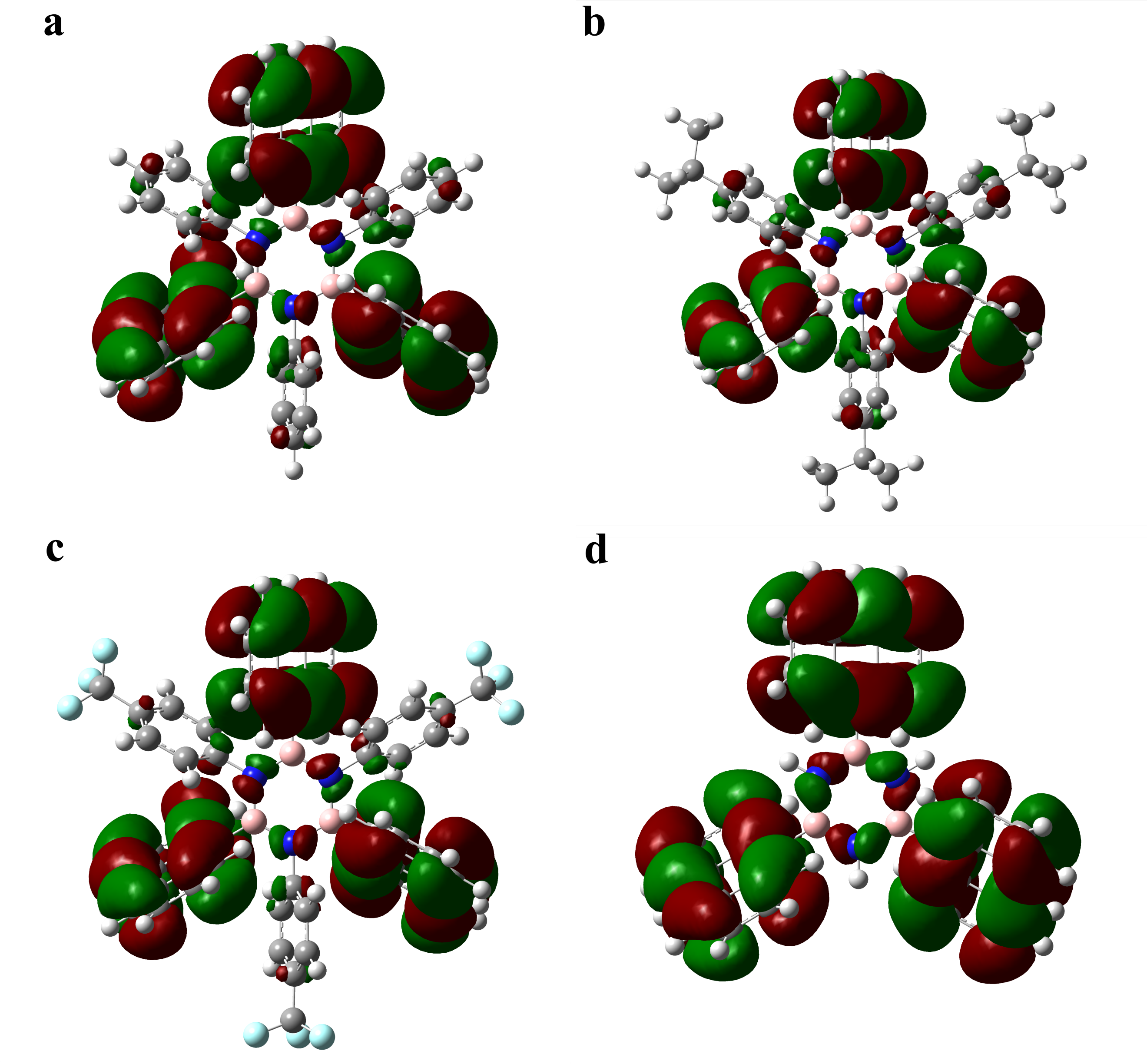


**Figure S2.** **a**, **b**, **c**, and **d**, Geometry optimized monomer configurations and pictorial representations of the KS HOMO for each type of borazine **1**, **2**, **3**, and **4**, respectively, with isosurface value = 0.01 (Figure 1 in the main text). In ball and stick, the molecular structures, with hydrogens in white, boron in pink, carbon in grey, nitrogen in blue, and fluorine in cyan, respectively.


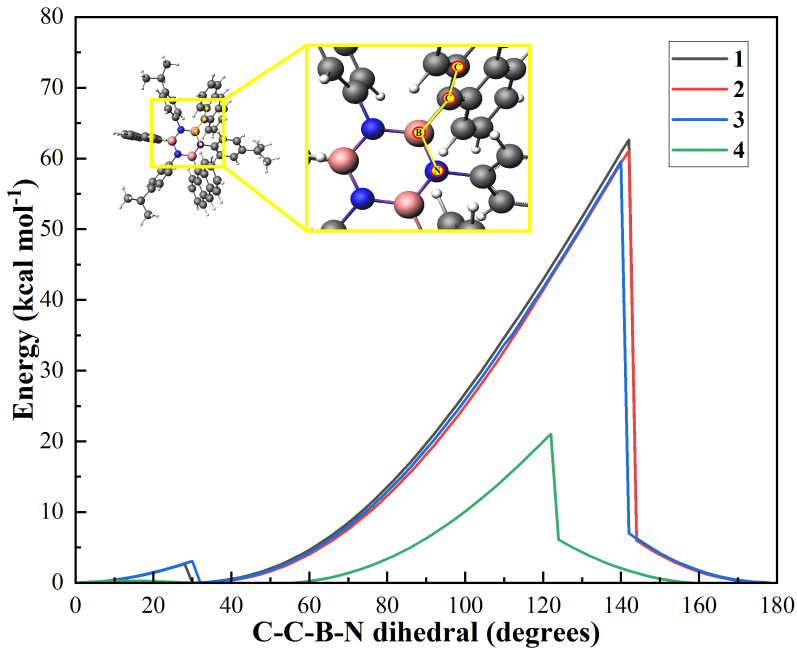


**Figure S3.** DFT energy scan (kcal mol^-1^) along the same C-C-B-N dihedral angle (degrees) for each borazine **1**, **2**, **3**, and **4**, in black, red, blue, and green lines, respectively. The yellow inset shows the representative C-C-B-N dihedral angle for all borazines.

**
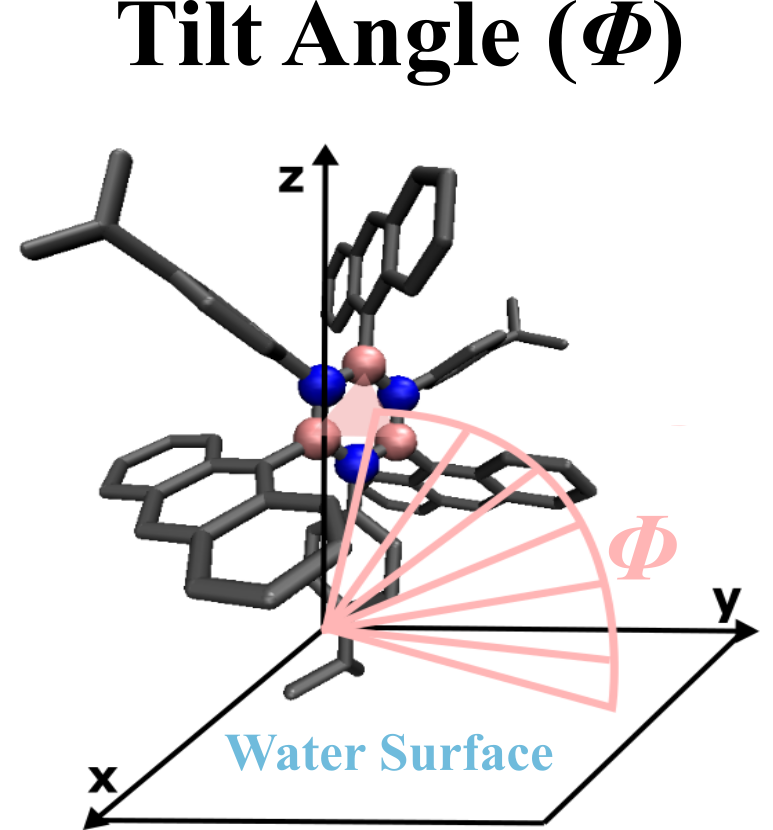
**

**Figure S4.** The definition of the tilt angle (*Φ*) as the arc between the plane of the borazine center of each borazine (pink) and the *x*-*y* plane of the water surface is illustrated. Borazine **2** is shown in balls and sticks as a representative case: boron, carbon, and nitrogen, are colored in pink, grey, and blue, respectively; hydrogens are omitted for clarity.


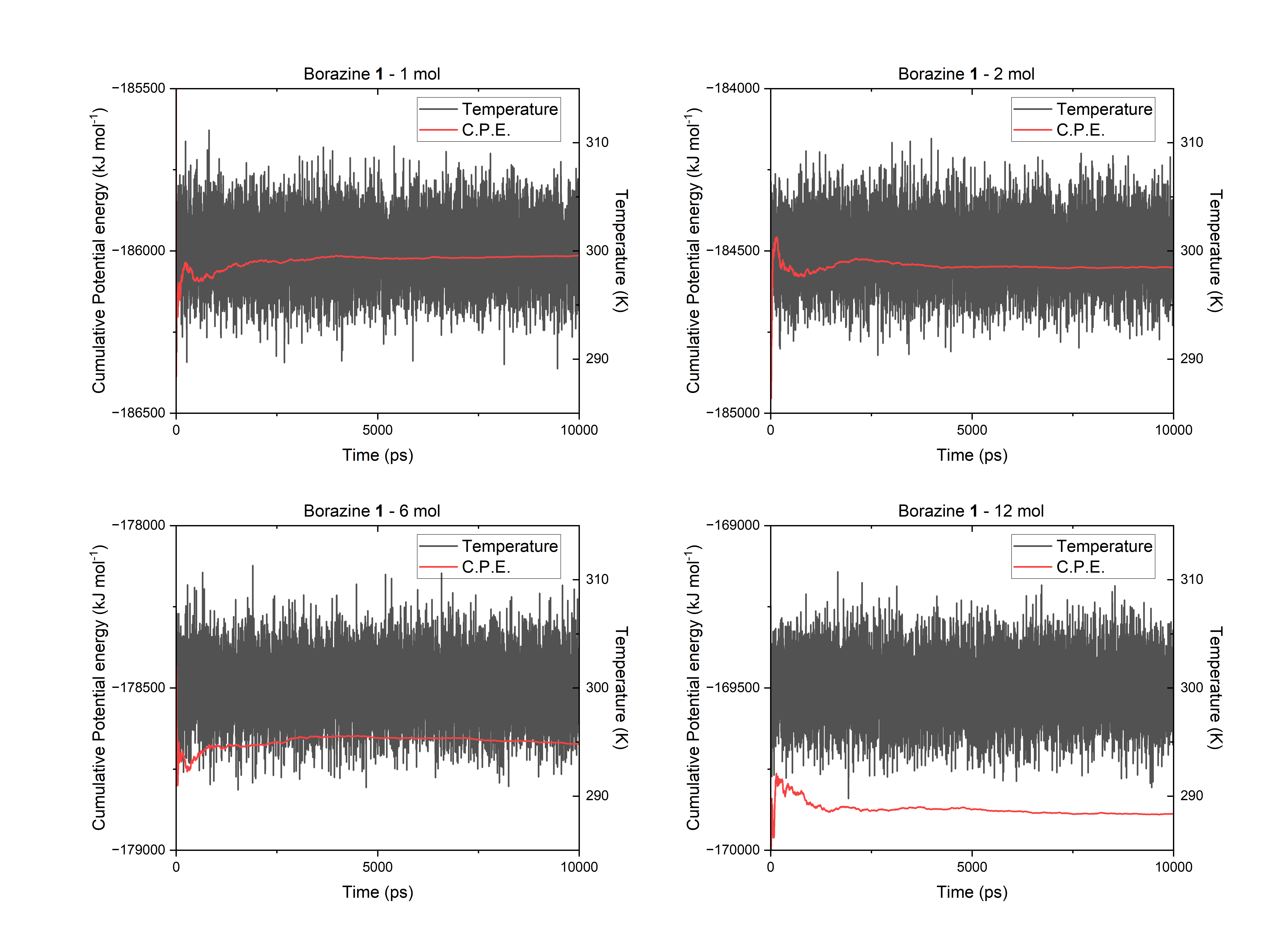


**
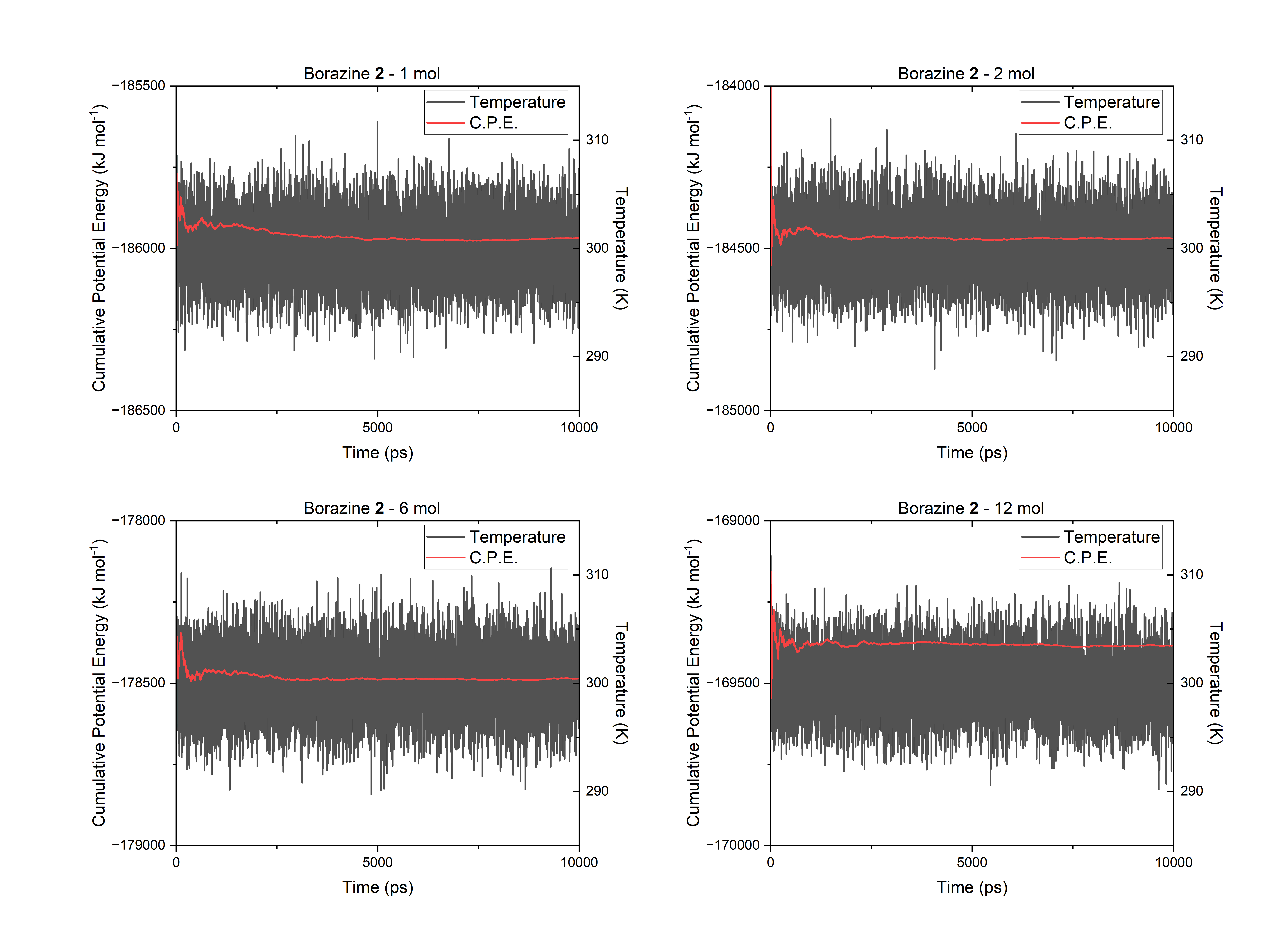
**

**
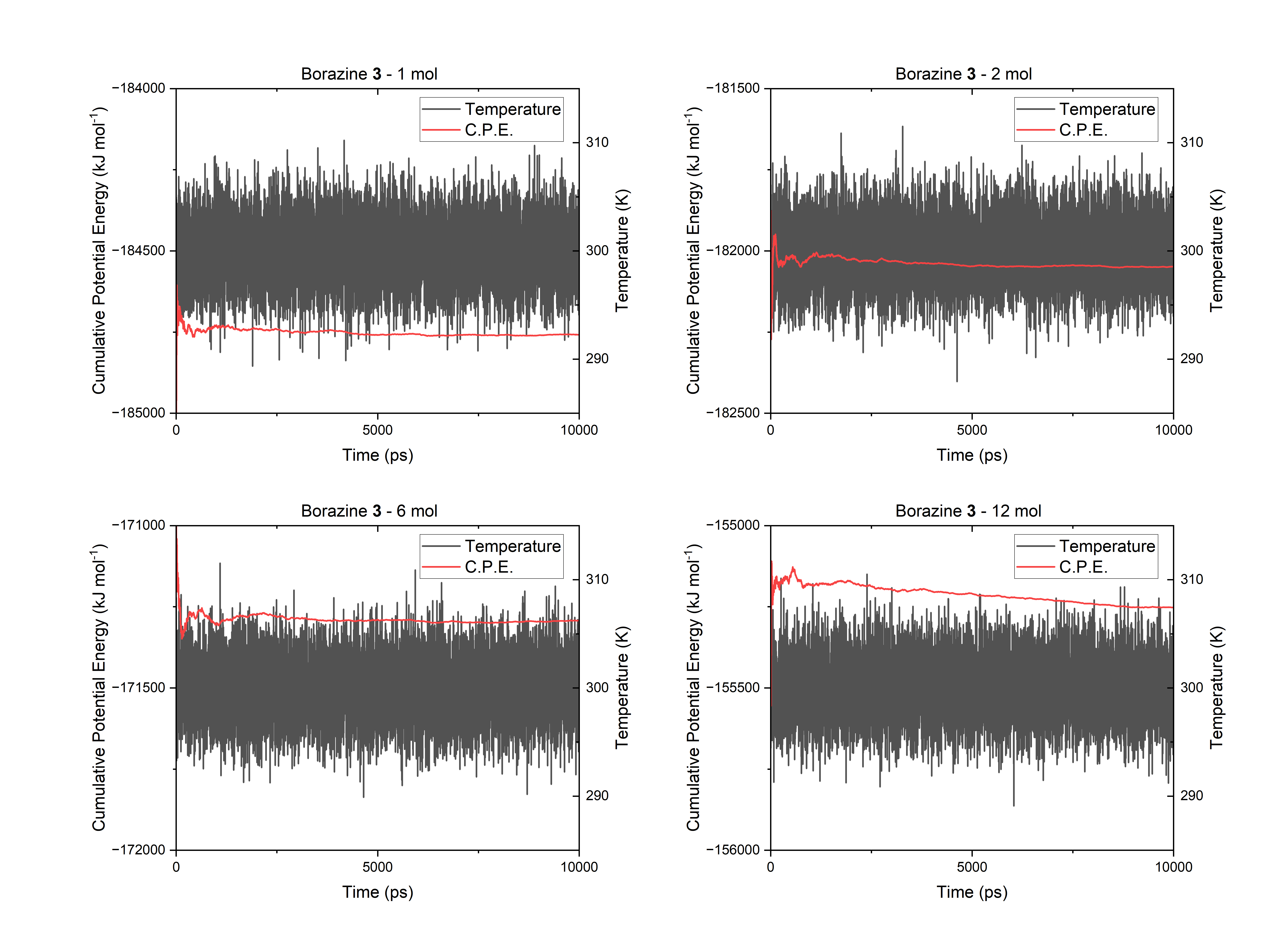
**

**
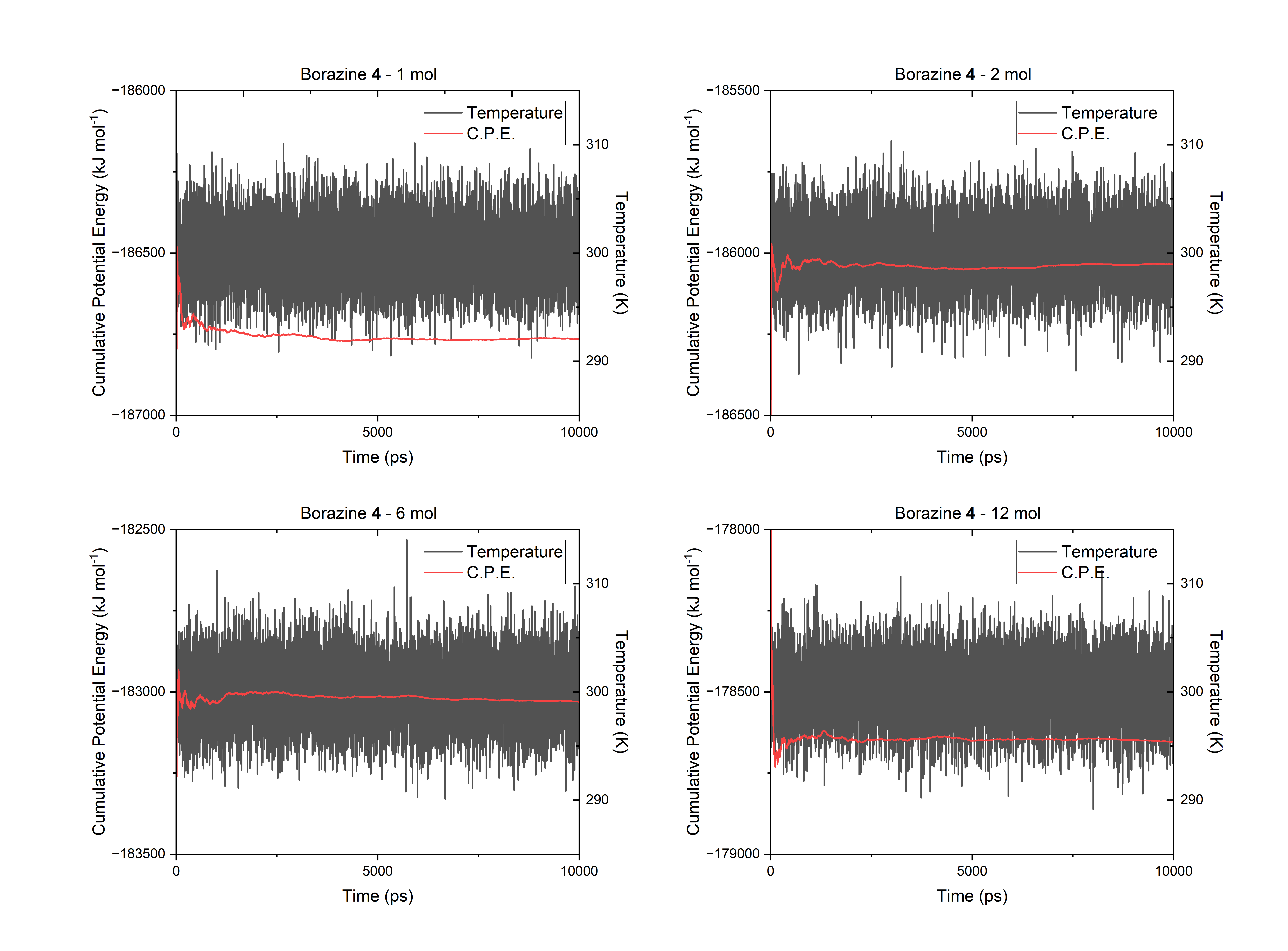
**

**Figure S5.** Temperature in K with black line, and cumulative potential energy (C.P.E.) in kJ mol^-1^ with red line, along the last 10 ns of the NVT MD simulations at 300 K per each borazine system **1**, **2**, **3**, and **4**, respectively, as derived from the borazine’s systems with 1, 2, 6, and 12 molecules (mol).

**S3. Synthesis of Borazine 2**

**S3.1. Synthesis of B,B′,B′′-Tri(9-anthryl)-N,N′,N′′-tris(p-isopropyl-phenyl) borazine (borazine 2) and bisanthryl-triisopropylphenyl borazine (borazine 5), and Elemental Analysis**

Chemical reagents were purchased from commercial suppliers and used without further purification. Solvents were dried over 4 Å mol sieves and purged with Argon before use. Flash column chromatography was performed on silica gel 60 Å (0.04 – 0.063 mm, Screening Devices B.V.). Thin Layer Chromatography (TLC) was performed on TLC Silica gel 60 plates (Kieselgel F254, Merck). Liquid-state NMR spectra were recorded on a Bruker AV-400WB NMR instrument. Chemical shifts are given in ppm (δ) relative to the solvent signals (CDCl_3_). Electrospray ionization mass spectra (ESI-MS) were obtained with a Thermo Scientific LCQ Fleet spectrometer equipped with an electrospray ion source (ESI) in positive ion mode. High-resolution mass spectra (HR-MS) were obtained with a Waters Synapt G2-Si (TOF) equipped with an ESI source in positive ion mode (source voltage 3.5 kV) and enkephalin (LeuEnk) as internal lock mass (M+H^+^ = 556.2771). Elemental analysis was performed by Mikroanalytisches Labor Kolbe (c/o Fraunhofer Institut UMSICHT).

**Figure S6**. Sketch structures of borazine **2** and **5**, on the left and right, respectively.

**S3.2. Experimental details borazine 2**

The experimental procedure can be found in the main text. ^1^H NMR (400 MHz, CDCl_3_) δ 8.39 (dd, J = 8.6, 1.0 Hz, 6H), 7.94 (s, 3H), 7.68 (dd, J = 8.0, 0.9 Hz, 6H), 7.55 (ddd, J = 8.6, 6.6, 1.3 Hz, 6H), 7.30 (ddd, J = 8.4, 6.6, 1.1 Hz, 9H), 6.51 (d, J = 8.4 Hz, 6H), 5.77 (d, J = 8.3 Hz, 6H), 2.00 (heptet, J = 6.9 Hz, 3H), 0.46 (d, J = 6.9 Hz, 18H). ^13^C NMR (101 MHz, CDCl_3_) δ 144.0, 142.8, 137.7, 133.1, 130.8, 129.6, 128.6, 126.6, 126.3, 124.43, 124.36, 123.9, 32.8, 23.3. ESI MS m/z 963.5 (M^+^). HR-MS m/z 964.5, 981.5 (M+H^+^, M+NH_4_^+^). Anal. Calcd for C_69_H_60_B_3_N_3_: C, 86.00; H, 6.28; N, 4.36. Found: C, 85.91; H, 6.29; N, 4.34.


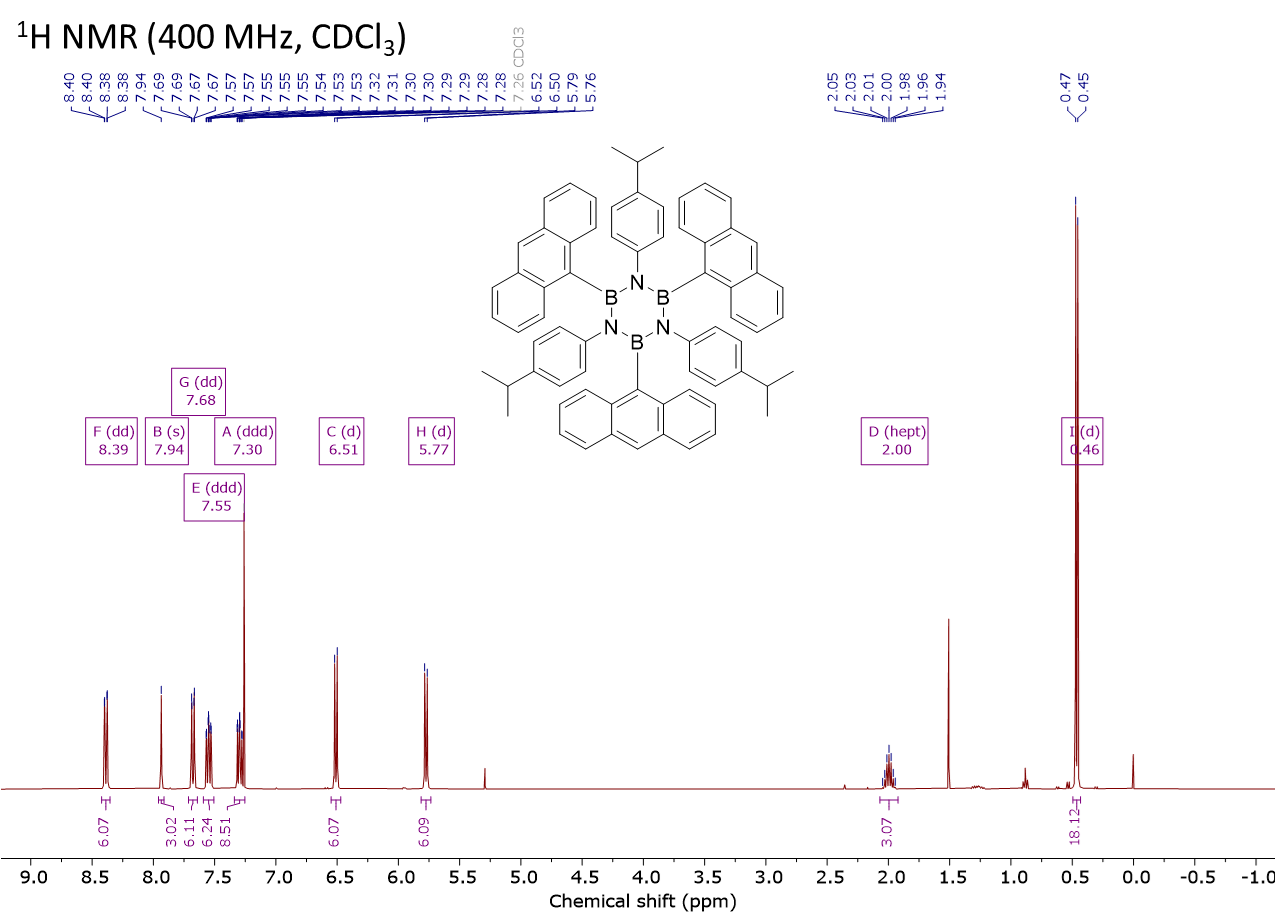


**Figure S7**. ^1^H NMR spectrum of borazine **2** in CDCl_3_.


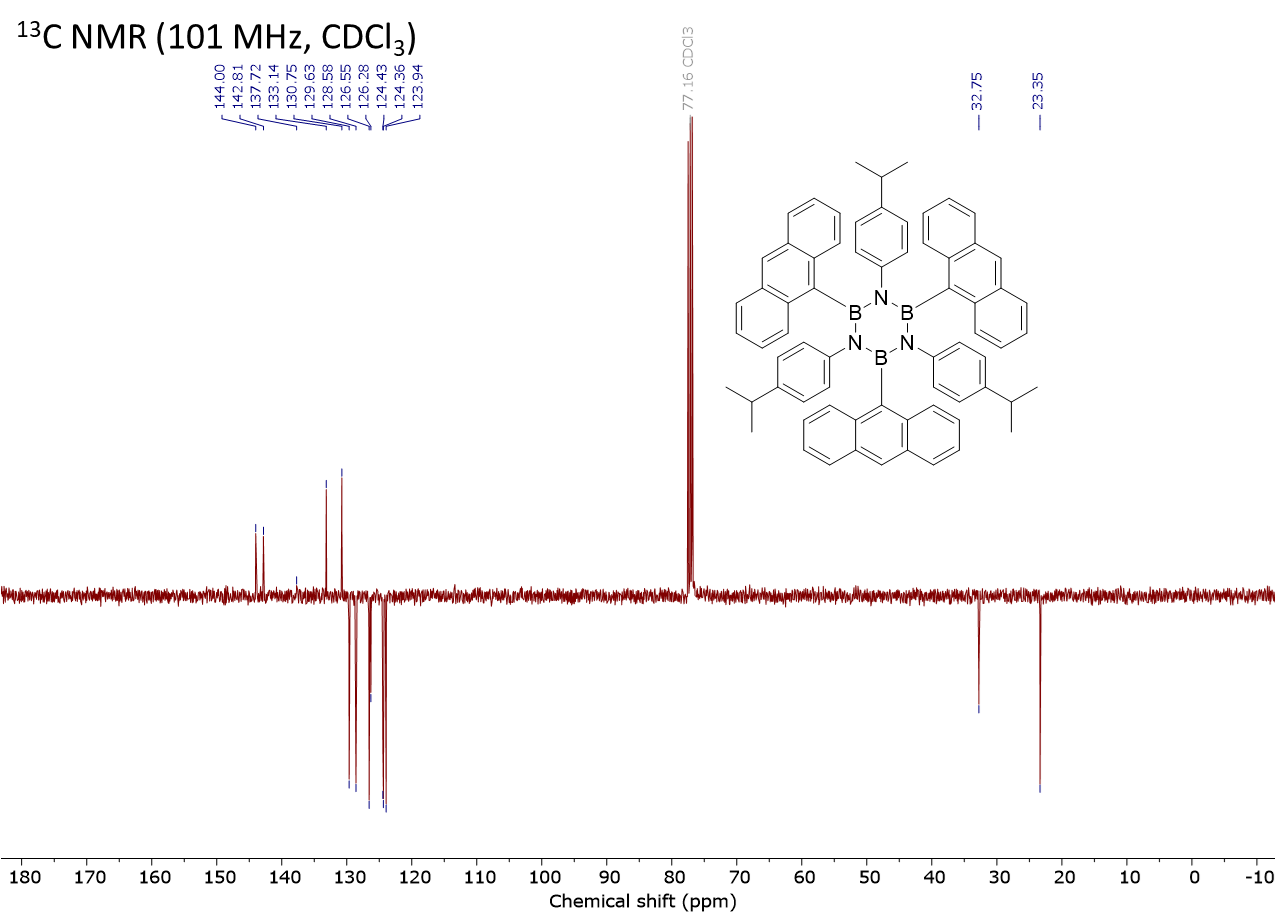


**Figure S8.** ^13^C NMR spectrum of borazine **2** in CDCl_3_.

**S3.3. Experimental details borazine 5**

Following the same experimental procedure as for borazine **2**, the bisanthryl-substituted borazine **5** was synthesized and purified (Rf = 0.5 in DCM/pentane 3:7) as yellow crystals (75 mg, 0.093 mmol, 28%). ^1^H NMR (400 MHz, CDCl_3_) δ 8.27 (dd, J = 8.7, 1.1 Hz, 4H), 7.93 (s, 2H), 7.67 (dd, J = 8.0, 1.2 Hz, 4H), 7.49 (ddd, J = 8.6, 6.6, 1.3 Hz, 4H), 7.29 (ddd, J = 8.4, 6.5, 1.1 Hz, 4H), 6.96 (d, J = 8.3 Hz, 4H), 6.62 (d, J = 8.3 Hz, 4H), 6.32 (d, J = 8.4 Hz, 2H), 5.66 (d, J = 8.4 Hz, 2H), 3.81 (s, 1H), 2.50 (heptet, J = 6.9 Hz, 2H), 1.93 (heptet, J = 6.8 Hz, 1H), 0.91 (d, J = 6.9 Hz, 12H), 0.41 (d, J = 6.9 Hz, 6H). ^13^C NMR (101 MHz, CDCl_3_) δ 145.4, 143.7, 142.7, 141.0, 137.4, 133.1, 130.7, 129.6, 128.5, 127.1, 126.6, 126.2, 126.1, 124.38, 124.35, 123.7, 33.2, 32.7, 23.7, 23.3. ESI MS m/z 803.4 (M^+^). HR-MS m/z 804.4, 821.5 (M+H^+^, M+NH_4_^+^). Anal. Calcd for C_55_H_52_B_3_N_3_∙5H_2_O: C, 73.93; H, 6.99; N, 4.70. Found: C, 73.18; H, 6.61; N, 4.62.


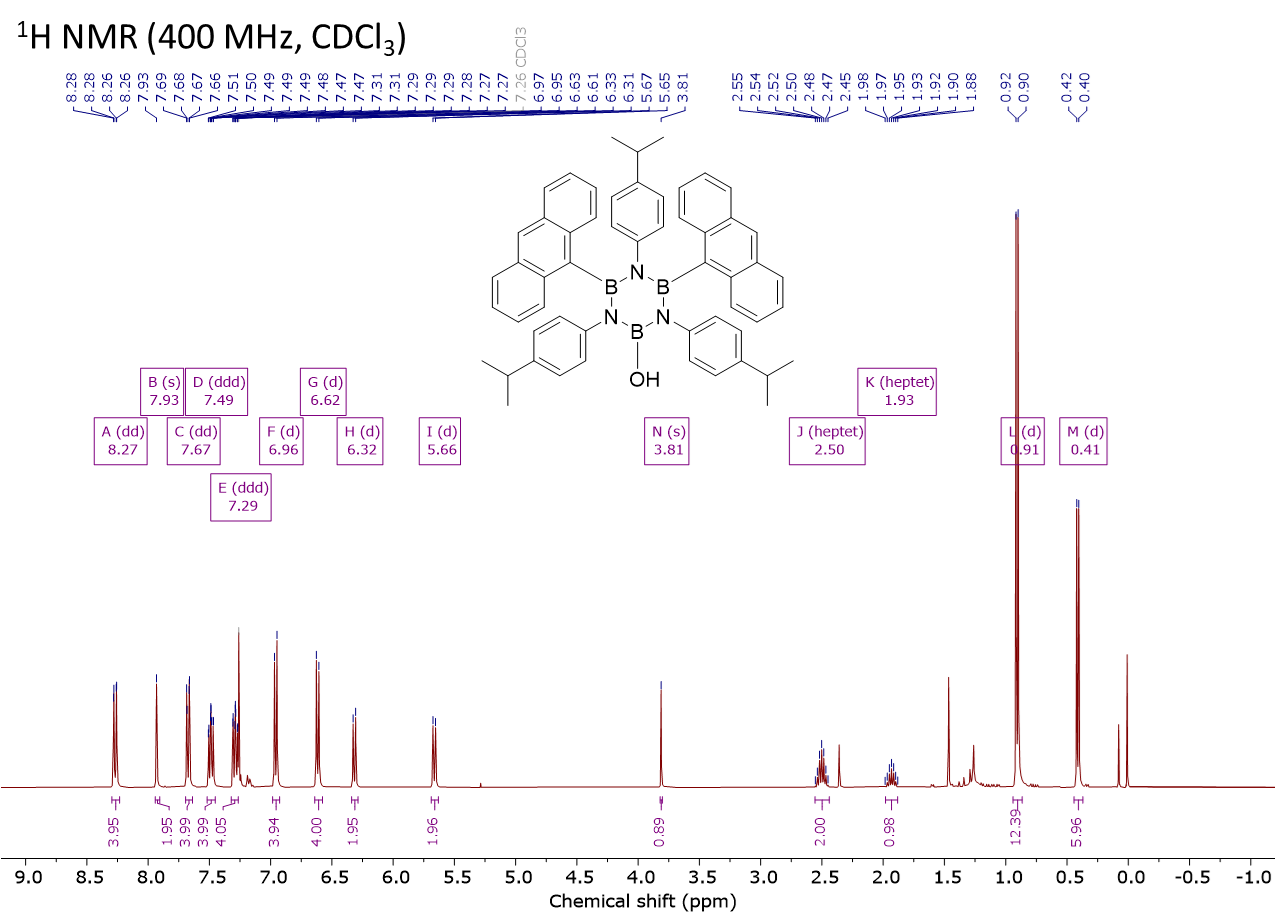


**Figure S9.** ^1^H NMR spectrum of borazine **5** in CDCl_3_.


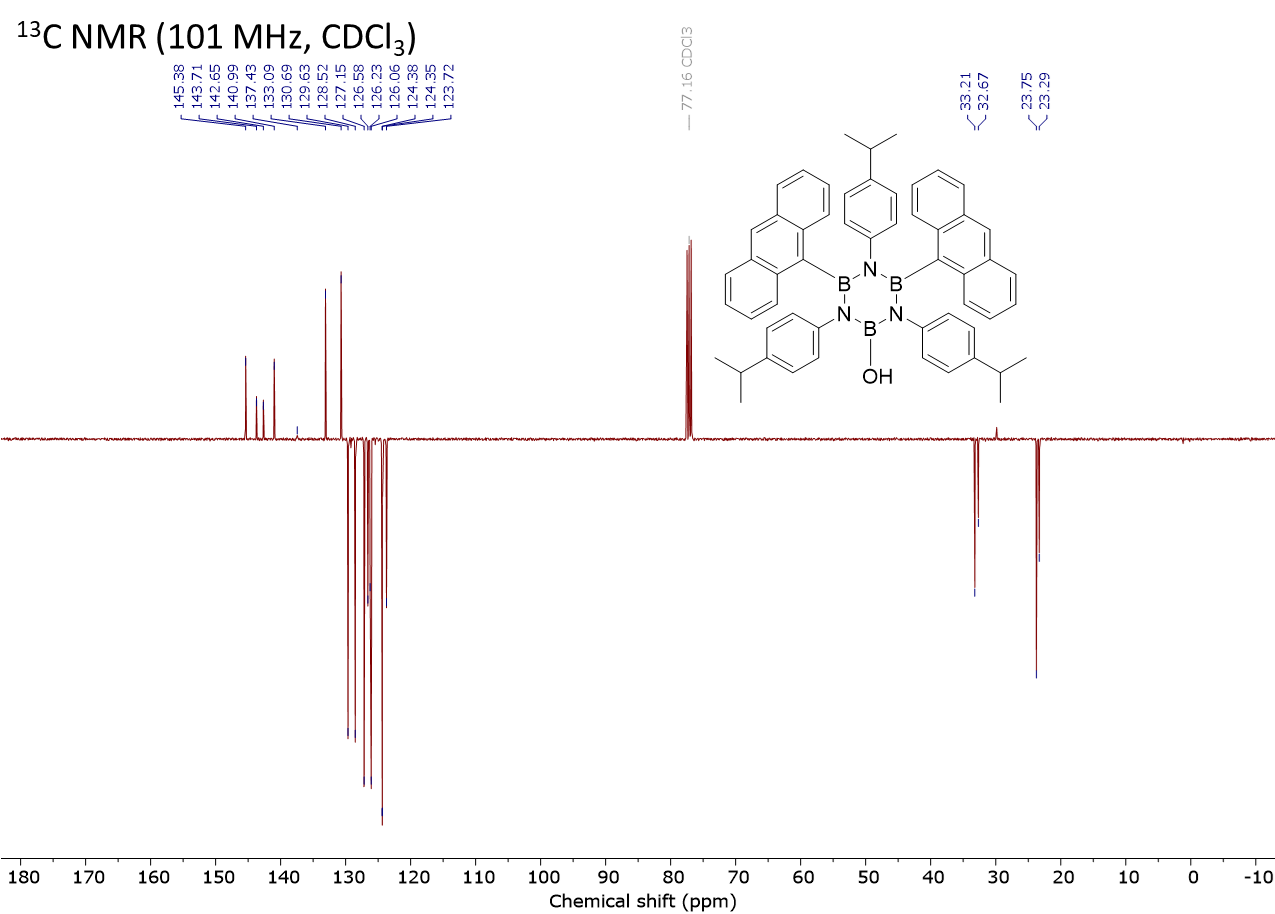


**Figure S10.** ^13^C NMR spectrum of borazine **5** in CDCl_3_.

**S4. Characterization of Borazine 2**

**S4.1. Single Crystal X-ray Crystallography of [borazine 2 (toluene)]**

**2_203K**: All reflection intensities were measured at 203(2) K* using a SuperNova diffractometer (equipped with Atlas detector) with Mo *K*α radiation (λ = 0.71073 Å) under the program CrysAlisPro (Version CrysAlisPro 1.171.42.49, Rigaku OD, 2022). The same program was used to refine the cell dimensions and for data reduction. The structure was solved with the program SHELXS-2018/3 (Sheldrick, 2018), and was refined on *F^2^* with SHELXL-2018/3 (Sheldrick, 2018).^1^ Numerical absorption correction based on gaussian integration over a multifaceted crystal model was applied using CrysAlisPro. The temperature of the data collection was controlled using the system Cryojet (manufactured by Oxford Instruments). The H atoms were placed at calculated positions using the instructions AFIX 13, AFIX 33, AFIX 43 or AFIX 137 with isotropic displacement parameters having values 1.2 or 1.5 *U*_eq_ of the attached C atoms. The structure is partly disordered. Two of the three 4-isopropylphenyl groups and the lattice toluene solvent molecule are disordered over two orientations, and the occupancy factors of the major components of the disorder refine to 0.700(7), 0.53(2) and 0.502(8).

**1_110K**: All reflection intensities were measured at 110 K** using a SuperNova diffractometer (equipped with Atlas detector) with Cu *K*α radiation (λ = 1.54178 Å) under the program CrysAlisPro (Version CrysAlisPro 1.171.42.49, Rigaku OD, 2022). The same program was used to refine the cell dimensions and for data reduction. The structure was solved with the program SHELXS-2018/3 (Sheldrick, 2018) and was refined on *F^2^* with SHELXL-2018/3 (Sheldrick, 2018).^1^ Analytical numeric absorption correction using a multifaceted crystal model was applied using CrysAlisPro. The temperature of the data collection was controlled using the system Cryojet (manufactured by Oxford Instruments). The H atoms were placed at calculated positions using the instructions AFIX 13, AFIX 33, AFIX 43 or AFIX 137 with isotropic displacement parameters having values 1.2 or 1.5 *U*_eq_ of the attached C atoms. The structure is mostly ordered. The lattice toluene solvent molecule is disordered over two orientations, and the occupancy factor of the major component of the disorder refines to 0.783(3). The reduced triclinic unit (*a* = 10.04 Å, *b* = 15.59 Å, *c* = 20.39 Å, *α* = 69.68°, *β* = 87.15°, *γ* = 83.53°) was transformed using the transformation matrix ***T*** = **(1 0 0 / 0 -1 0 / 0 1 -1)** to that the transformed unit cell of the low-temperature phase (**2_110K**) have comparable settings with that of the higher-temperature phase (**2_203K**).

* Any attempts to flash-cool the crystals from room temperature to 110 K and 150 K were unsuccessful as the crystals suffered from significant crystal damage (the crystals shattered very quickly, and diffraction was very poor), which is due to a solid-solid phase transition. When another crystal was flash-cooled from room temperature to 203 K, the crystal quality remained intact, and the diffraction pattern was consistent with that of a single crystal.

** The same crystal that was used for **2_203K** has been used here for **2_110K**. After a full data set was collected at 203 K, the crystal was allowed to be slowly cooled down from 203 to 110 K in 35-45 min. At 110 K, the crystal is clearly colorless (and yellow at 203K). Throughout the cooling process, the crystal slightly cracked but the long-range order of the crystal remained mostly intact as the crystal diffracted well at higher resolution.

**Table S1.** Crystallographic Data of 2_203K, CCDC 2409120 (<https://www.ccdc.cam.ac.uk/structures/Search?access=referee&ccdc=2409120&Author=D.+Calvani>)

|  | **2_203K** |
| --- | --- |
| Crystal data | |
| Chemical formula | C_69_H_60_B_3_N_3_·C_7_H_8_ |
| *M*_r_ | 1055.76 |
| Crystal system, space group | Triclinic, *P*-1 |
| Temperature (K) | 203 |
| *a*, *b*, *c* (Å) | 10.0141 (4), 15.7151 (9), 20.9899 (8) |
| α, β, γ (°) | 110.870 (4), 91.583 (3), 96.480 (4) |
| *V* (Å^3^) | 3058.6 (3) |
| *Z* | 2 |
| Radiation type | Mo *K*α |
| μ (mm^-1^) | 0.07 |
| Crystal size (mm) | 0.35 × 0.11 × 0.08 |
|  | |
| Data collection | |
| Diffractometer | SuperNova, Dual, Cu at zero, Atlas |
| Absorption correction | Gaussian  *CrysAlis PRO* 1.171.42.49 (Rigaku Oxford Diffraction, 2022) Numerical absorption correction based on gaussian integration over a multifaceted crystal model Empirical absorption correction using spherical harmonics, implemented in SCALE3 ABSPACK scaling algorithm. |
| *T*_min_, *T*_max_ | 0.658, 1.000 |
| No. of measured, independent and  observed [*I* > 2σ(*I*)] reflections | 45752, 10743, 6934 |
| *R*_int_ | 0.060 |
| (sin θ/λ)_max_ (Å^-1^) | 0.595 |
|  | |
| Refinement | |
| *R*[*F*^2^ > 2σ(*F*^2^)], *wR*(*F*^2^), *S* | 0.062, 0.182, 1.04 |
| No. of reflections | 10743 |
| No. of parameters | 896 |
| No. of restraints | 871 |
| H-atom treatment | H-atom parameters constrained |
| Δρ_max_, Δρ_min_ (e Å^-3^) | 0.31, -0.28 |

Computer programs: *CrysAlis PRO* 1.171.42.49 (Rigaku OD, 2022), *SHELXS2018*/3 (Sheldrick, 2018), *SHELXL2018*/3 (Sheldrick, 2018), *SHELXTL* v6.10 (Sheldrick, 2008).^1^

**Table S2.** Crystallographic Data of 1_110K, CCDC 2409119 (<https://www.ccdc.cam.ac.uk/structures/Search?access=referee&ccdc=2409119&Author=D.+Calvani>)

|  | **1_110K** |
| --- | --- |
| Crystal data | |
| Chemical formula | C_69_H_60_B_3_N_3_·C_7_H_8_ |
| *M*_r_ | 1055.76 |
| Crystal system, space group | Triclinic, *P*-1 |
| Temperature (K) | 110 |
| *a*, *b*, *c* (Å) | 10.03783 (18), 15.5912 (3), 20.9249 (4) |
| α, β, γ (°) | 113.9996 (18), 87.9640 (15), 96.4719 (15) |
| *V* (Å^3^) | 2972.42 (10) |
| *Z* | 2 |
| Radiation type | Cu *K*α |
| μ (mm^-1^) | 0.51 |
| Crystal size (mm) | 0.35 × 0.11 × 0.08 |
|  | |
| Data collection | |
| Diffractometer | SuperNova, Dual, Cu at zero, Atlas |
| Absorption correction | Analytical  *CrysAlis PRO* 1.171.42.49 (Rigaku Oxford Diffraction, 2022) Analytical numeric absorption correction using a multifaceted crystal model based on expressions derived by R.C. Clark & J.S. Reid. (Clark, R. C. & Reid, J. S. (1995). Acta Cryst. A51, 887-897) Empirical absorption correction using spherical harmonics, implemented in SCALE3 ABSPACK scaling algorithm. |
| *T*_min_, *T*_max_ | 0.866, 0.967 |
| No. of measured, independent and  observed [*I* > 2σ(*I*)] reflections | 43342, 11627, 10034 |
| *R*_int_ | 0.029 |
| (sin θ/λ)_max_ (Å^-1^) | 0.617 |
|  | |
| Refinement | |
| *R*[*F*^2^ > 2σ(*F*^2^)], *wR*(*F*^2^), *S* | 0.045, 0.131, 1.02 |
| No. of reflections | 11627 |
| No. of parameters | 804 |
| No. of restraints | 257 |
| H-atom treatment | H-atom parameters constrained |
| Δρ_max_, Δρ_min_ (e Å^-3^) | 0.49, -0.43 |

Computer programs: *CrysAlis PRO* 1.171.42.49 (Rigaku OD, 2022), *SHELXS2018*/3 (Sheldrick, 2018), *SHELXL2018*/3 (Sheldrick, 2018), *SHELXTL* v6.10 (Sheldrick, 2008).^1^


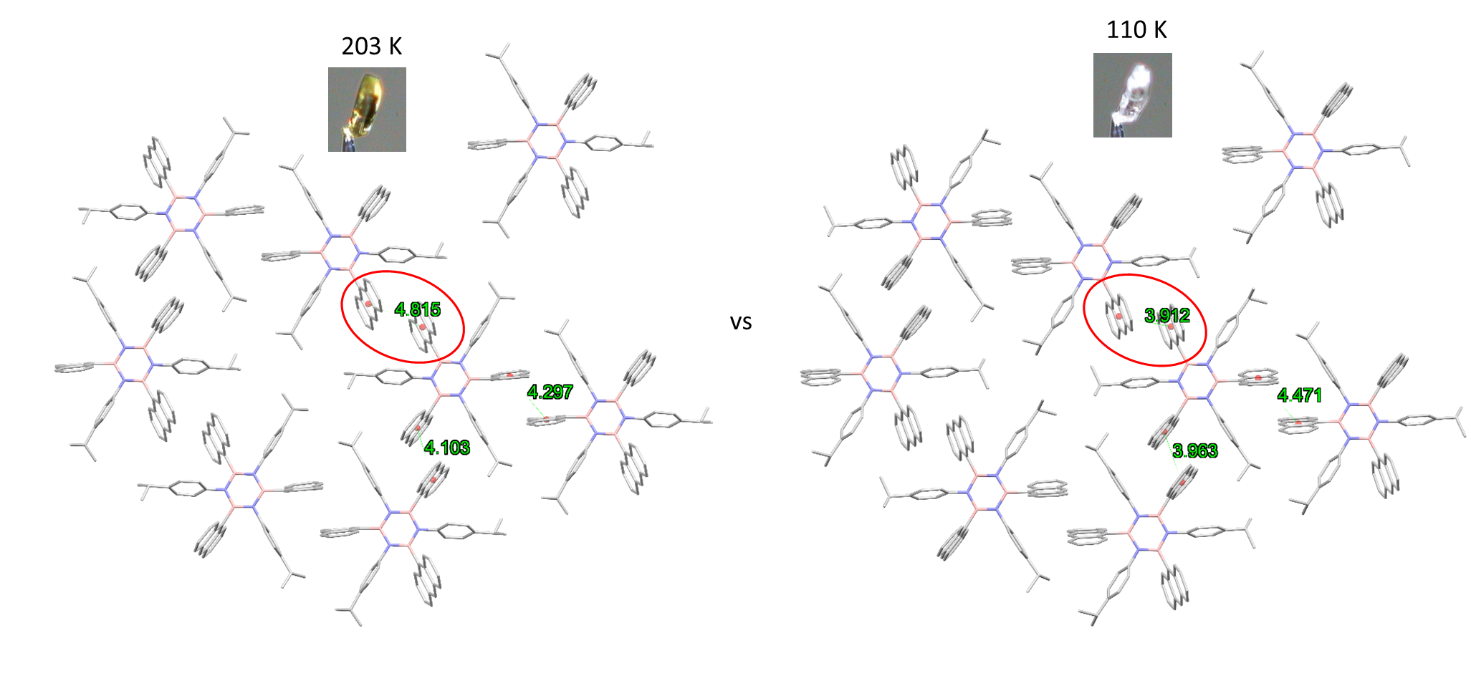


**Figure S11.** Crystal structures of borazine **2** obtained from slow diffusion of pentane into a solution of borazine **2** in DCM:toluene 1:1 (v/v). Upon slow cooling from 203 K to 110 K the distance between anthracene moieties decreases from 4.815 to 3.912 Å (circled in red) along with a color change of the crystals from yellow to white.

**S4.2. Liquid NMR and solid-state NMR (SS-NMR) borazine 2**

Liquid-state NMR spectra were recorded on Bruker Avance-III-HD 850 MHz standard bore liquid-state NMR spectrometer. Solid-state NMR spectra were recorded on a Bruker Neo console 750 MHz wide bore SS-NMR spectrometer. Chemical shifts are given in ppm (δ) relative to their respective residual solvent signals (CD_2_Cl_2_).


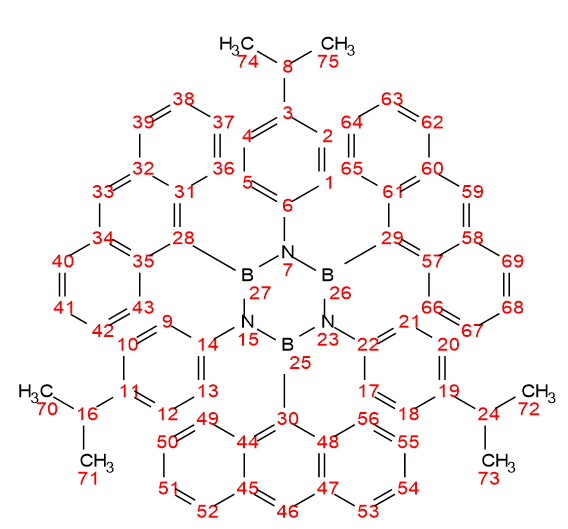


**Figure S12.** Structure of borazine **2** with atom numbers for NMR assignment.


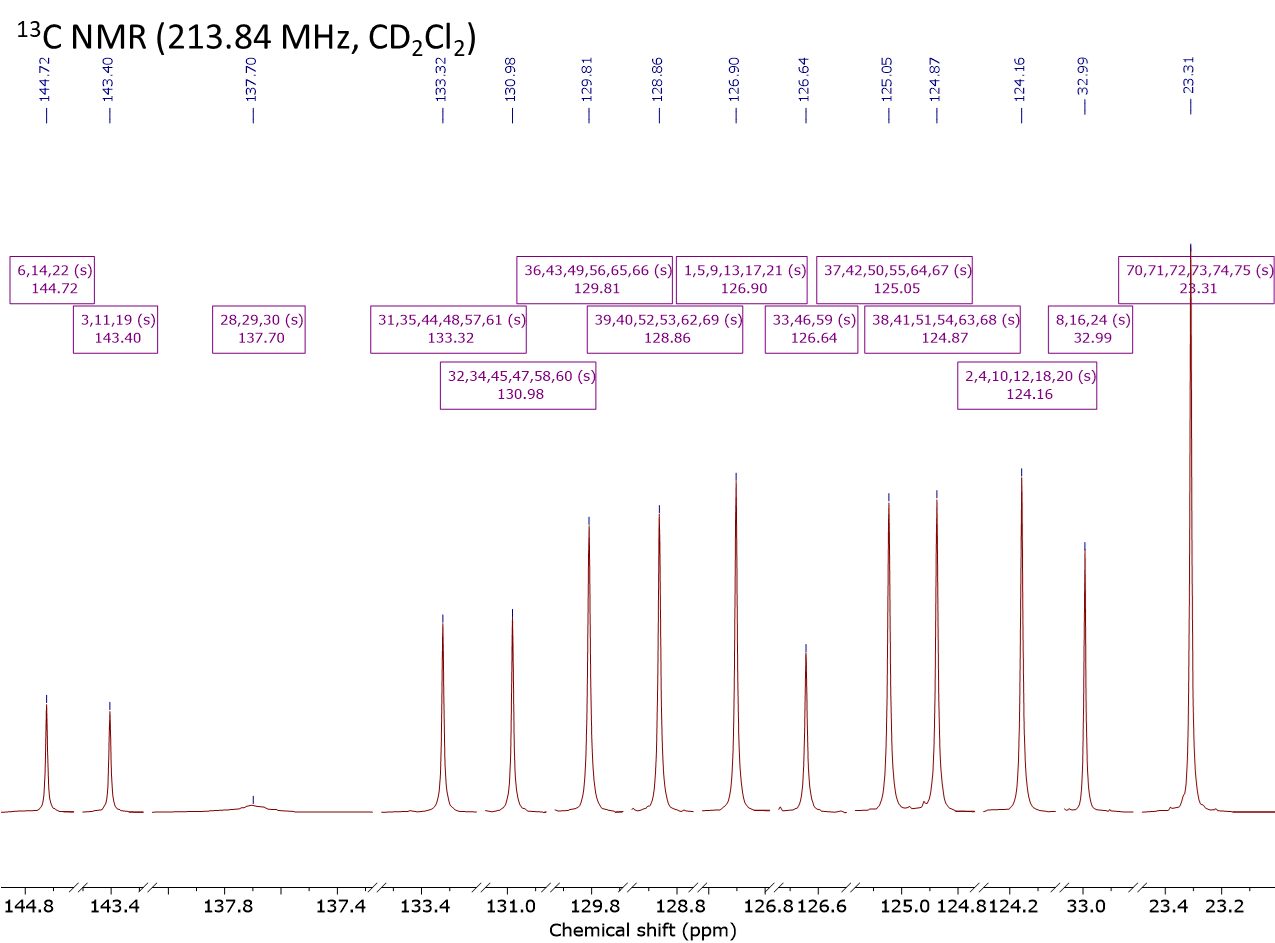


**Figure S13.** ^13^C NMR spectrum of borazine **2** in CD_2_Cl_2_.


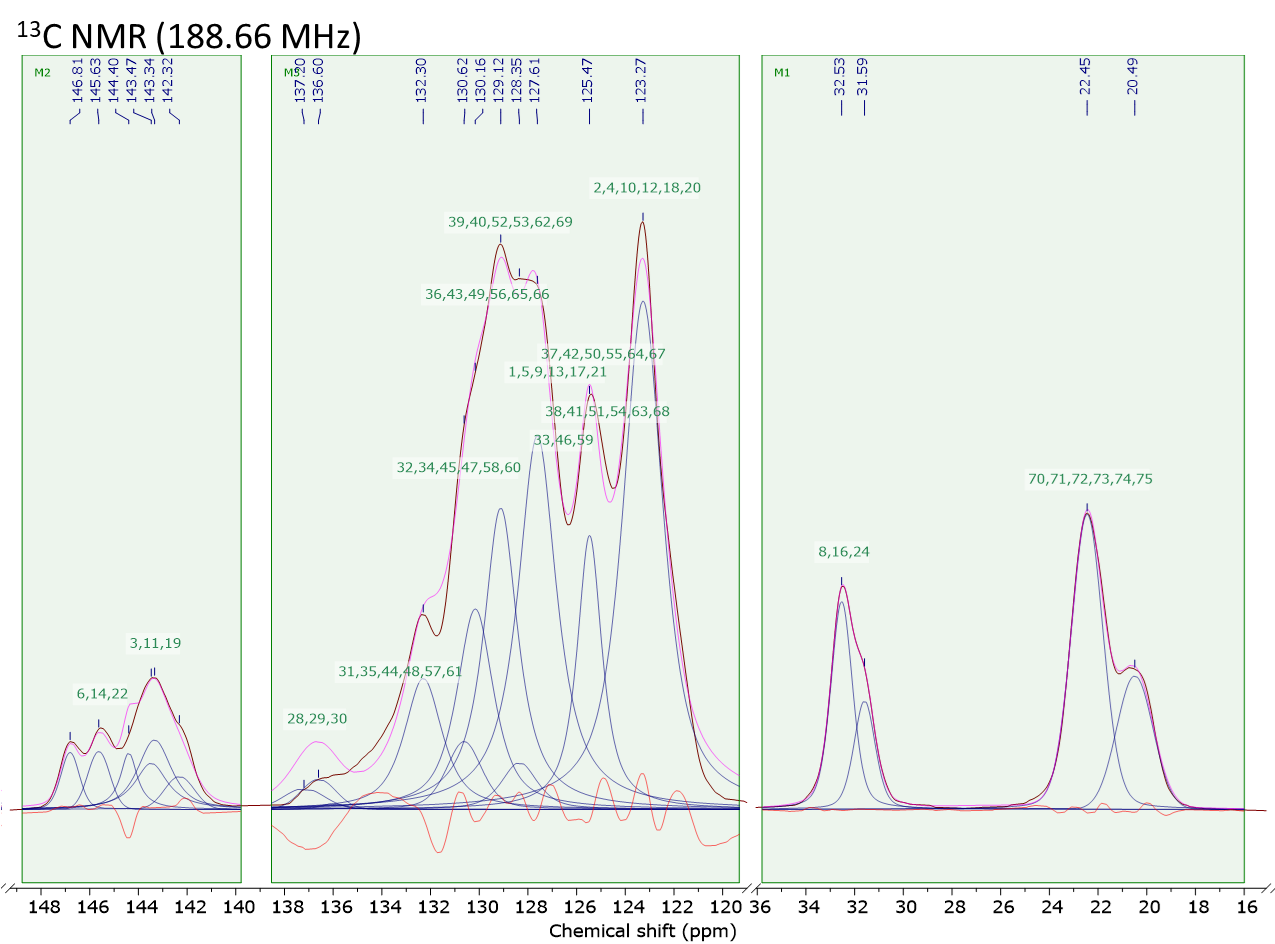


**Figure S14.** SS-NMR ^13^C data was collected from the borazine **2** at room temperature.


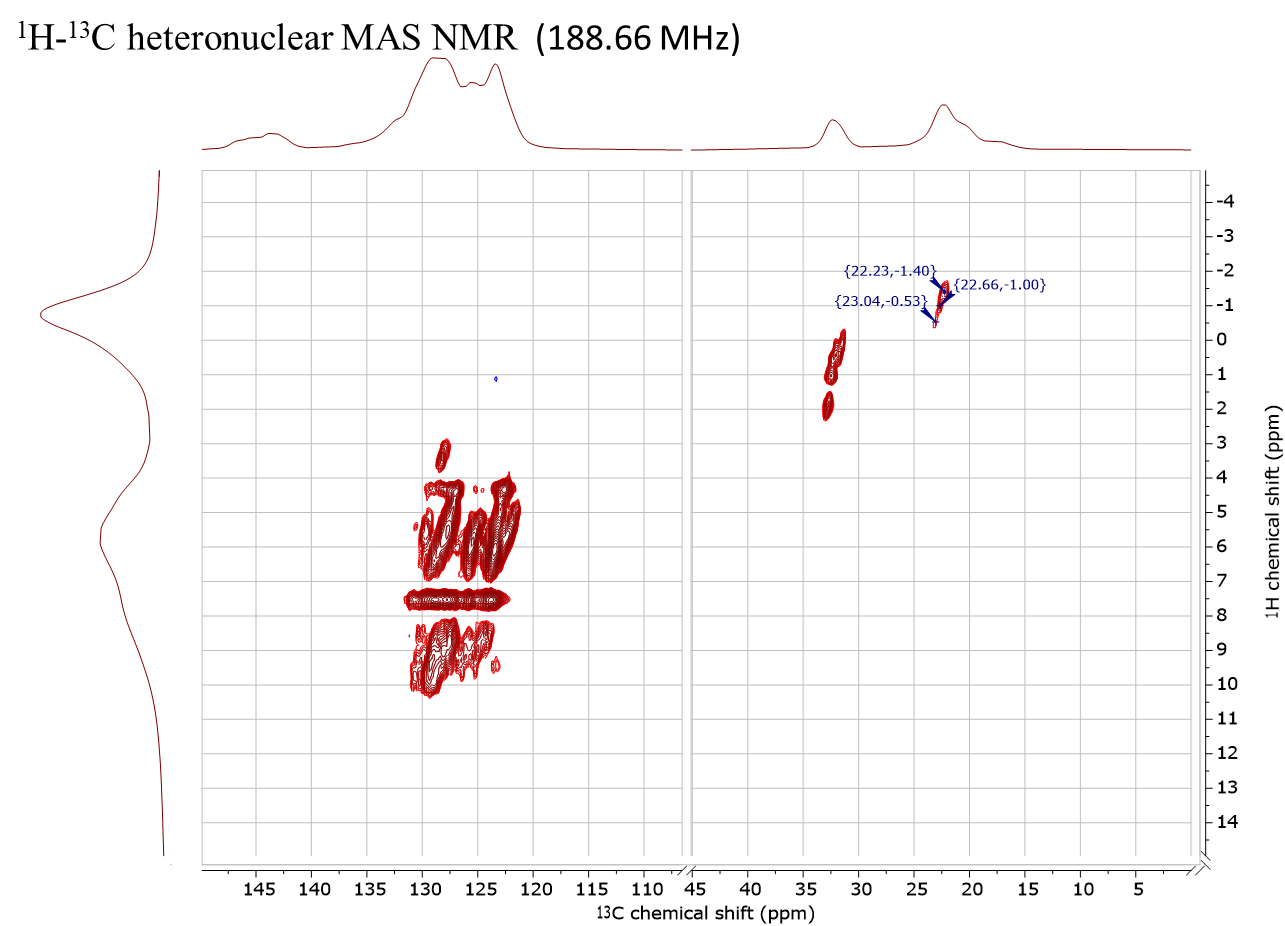


**Figure S15.** Contour plot sections of ^1^H-^13^C heteronuclear MAS NMR dipolar correlation spectra of borazine **2** were recorded at a field of 17.6 T, employing a spinning rate of 20 kHz and sample temperatures of 298 K. The assignments of the CH_2_ and CH_3_ isopropyl signals are indicated.

**S4.3. Comparison of experimental liquid NMR, solid-state NMR, and computational NMR**

**Table S3.** List of chemical shift values (in ppm) and assignments for borazine **2** in liquid NMR (in DCM), solid-state NMR (SS-NMR), and DFT (PBE-D3/TZP basis set) results for the monomer and dimer with standard deviation (STDEV) 2.44 ppm and 1.82 ppm, respectively.

**S5. Langmuir-Blodgett Film Fabrication, Langmuir-Blodgett-like MD Simulations, and Surface-Tension Results**

**S5.1. Spreading conditions at the water-to-air interface**

A KSV mini trough (surface area 243 cm^2^) equipped with a platinum Wilhelmy plate was used. The trough was made of Teflon® and the barriers of hydrophilic Delrin®. The subphase was Millipore water. For cleaning, the trough was first rinsed with Millipore water (3x), then wiped with chloroform:methanol 3:1 (v:v) with dust-free paper, and finally rinsed with Millipore water again. The barriers were wiped with Millipore water and chloroform with dust-free paper. The trough was then filled with Millipore water, and the surface was cleaned by aspiration to remove any residual particles. For spreading experiments, analytical grade chloroform (>99,9%, stabilized with amylene) was freshly distilled using clean glassware, stored in the dark, and used within 2 weeks. Distillation has a small effect on removing chloroform contamination at larger volumes, as the isotherm is shifted to higher MMAs at larger volumes (Figure S16). However, no significant differences in thin film properties were found from AFM and SEM analyses. Stock solutions of borazine **2** or POPC in chloroform were prepared at 1 mg mL^-1^ and then further diluted to the respective concentrations. Spreading was performed using a 100 µL air-tight Hamilton syringe equipped with a Hamilton Kel-F hub blunt point (26s gauge) needle. The solution was carefully spread at the interface by bringing the droplets hanging from the syringe into contact with the water surface at a rate of ~20 µL min^‑1^. The stock solutions were stored in the dark at 4 °C and used within 2 weeks. The compression was started 20 minutes after spreading to allow complete evaporation of the solvent at a speed of 2 mm min^-1^. After reaching the desired surface pressure, the Langmuir film was allowed to stabilize for 15 min before deposition onto a solid substrate. The average and standard deviation from multiple data sets of isotherms were calculated in OriginPro 2002 using the Average Multiple Curve; average following curve trace; 1000 points; and linear interpolation.

**
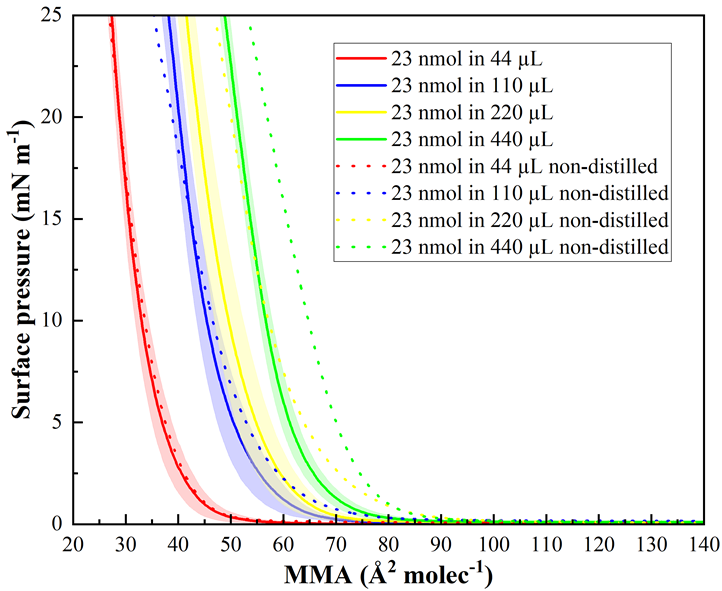
**

**Figure S16.** Effect of solvent distillation on the isotherms after depositing 23 nmol borazine **2** in increasing amounts of CHCl_3_, where the surface pressure in mN m^-1^ depends on the mean molecular area (MMA) in Å^2^ molec^-1^. At lower volumes, the additional solvent distillation has no effect. However, at higher volumes, the isotherms are shifted to higher mean molecular area (MMA) values for non-distilled chloroform, indicating that at higher volumes, solvent contamination could play a role.


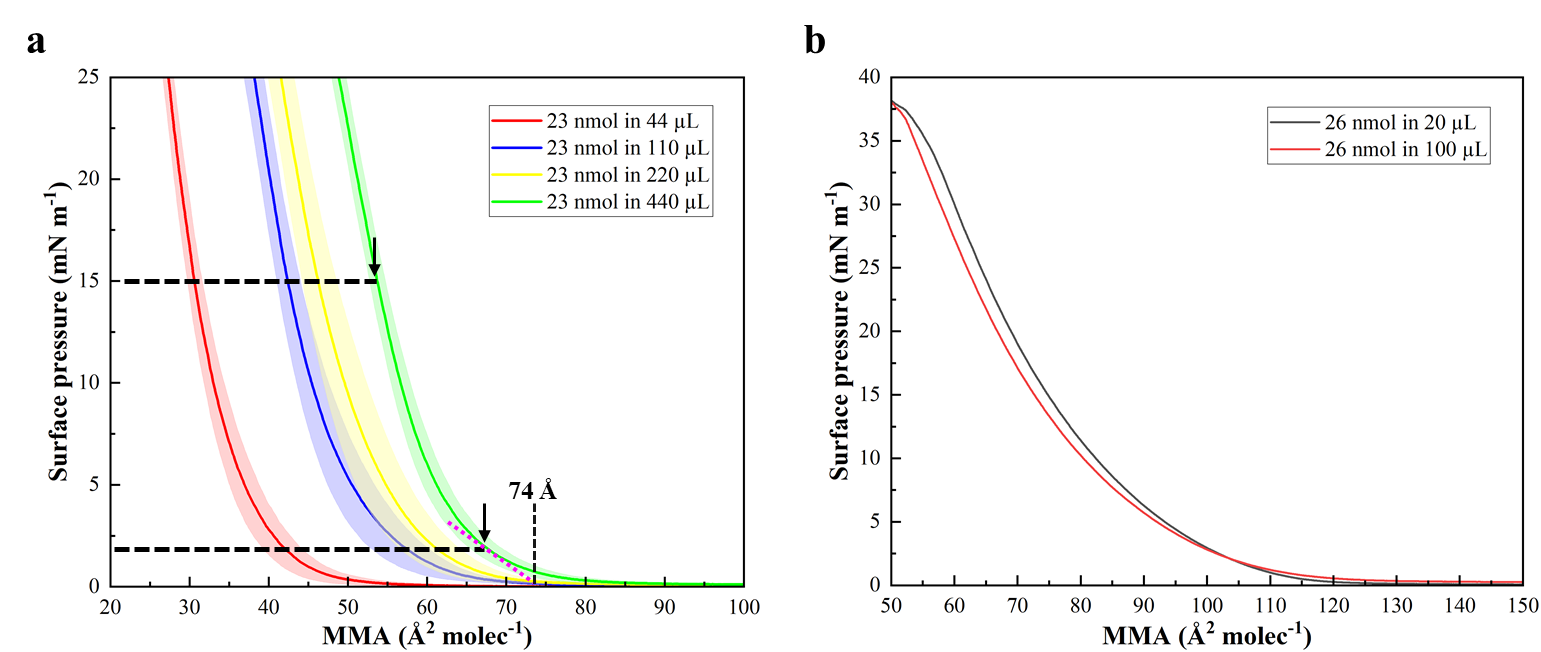


**Figure S17.** **a**, Langmuir–Blodgett isotherms of the borazine **2** system after depositing 23 nmol borazine **2** in increasing amounts of CHCl_3_, where the surface pressure in mN m^-1^ depends on the mean molecular area (MMA) in Å^2^ molec^-1^. Upon dilution, the isotherm is shifted to higher MMA values. The intersection of the extrapolated violet dashed line indicates the MMA at which the morphology that corresponds to the point of tangency (in this case 2 mN m^-1^) starts to take place if the film was perfectly distributed homogeneously. **b**, Isotherm of 26 nmol POPC in increasing amounts of CHCl_3_. Dilution does not affect the isotherm, indicating the ideal spreading behavior of POPC on the water surface during and after deposition.


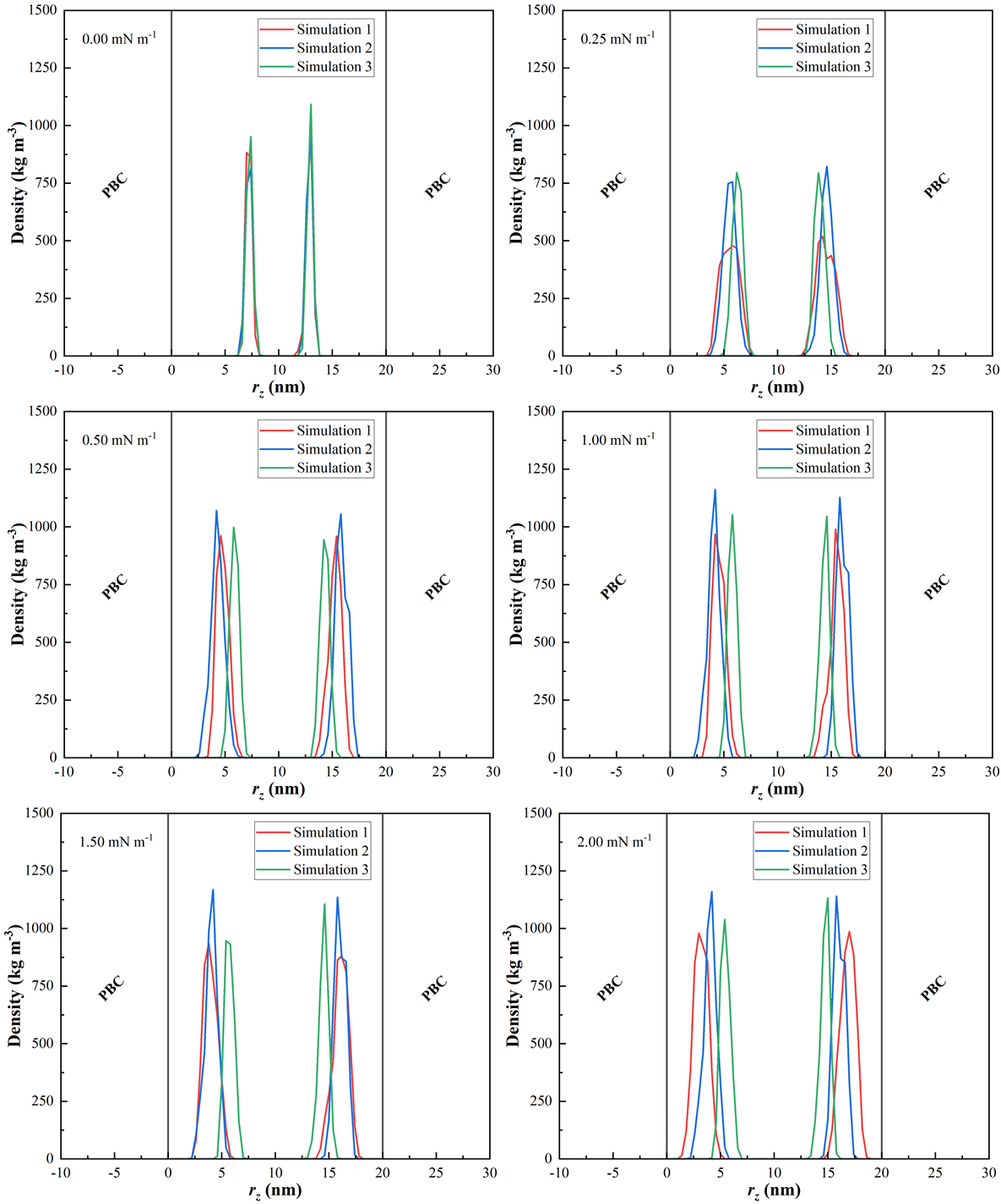


**Figure S18.** Density curve (kg m^-3^) of the borazine 2 molecules along the *z*-coordinate *r_z_* (nm) of the simulation box at different surface pressures (0, 0.25, 0.50, 1.00, 1.50, 2.00 mN m^-1^) for the three independent MD simulations, in blue, red, and green solid lines, respectively. The edges of the simulation box along the *z*-coordinate are indicated with the vertical solid black lines, and the rest is repeated by the periodic boundary conditions (PBC).


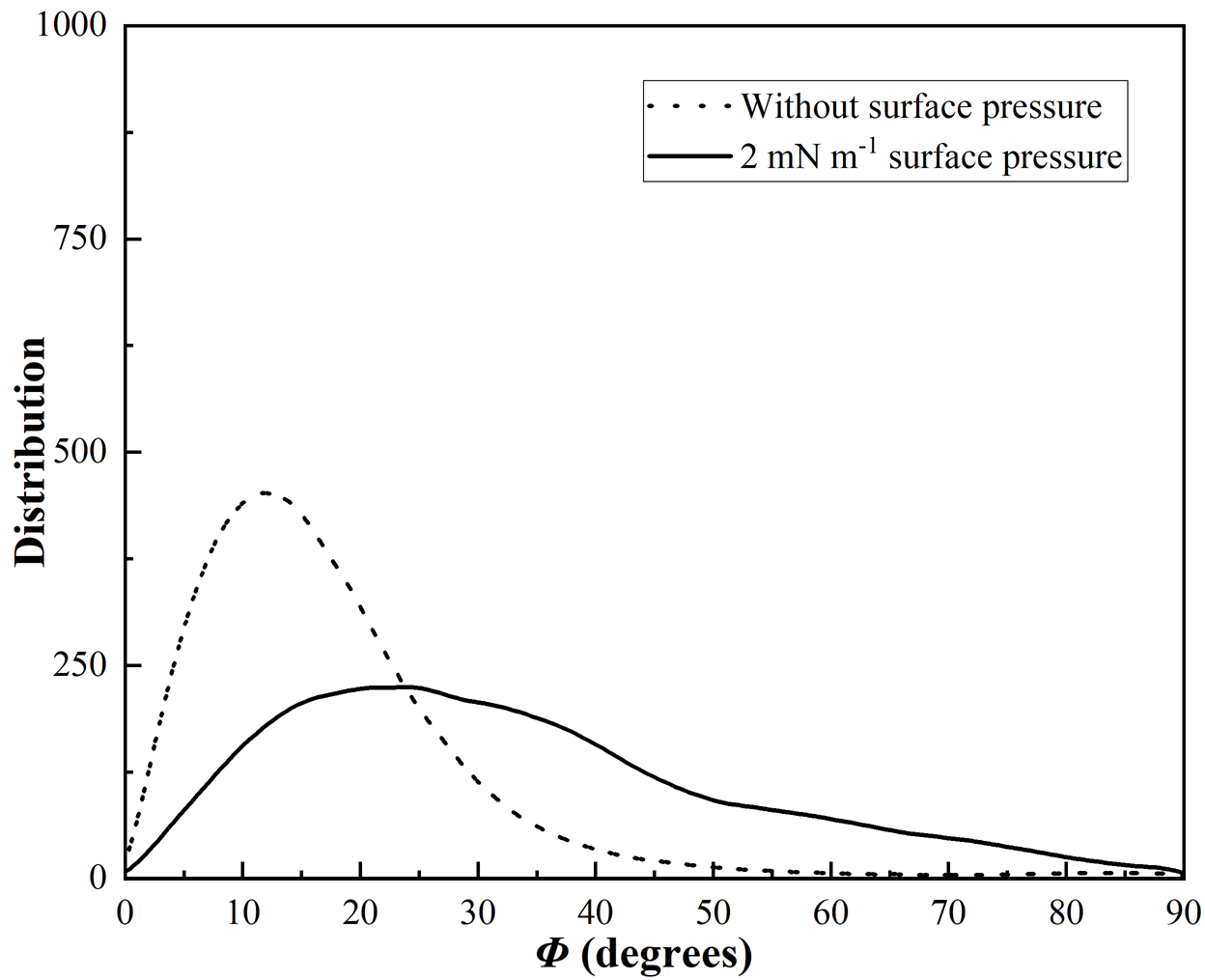


**Figure S19.** The plot shows the distribution of the tilt angle (*Φ*) of the 12 borazine **2** molecules system before compression at 300 K (without surface pressure - black dashed line), and surface pressure 2 mN m^-1^ and 300 K (2 mN m^-1^ surface pressure – black solid line), extracted from the average over the 3 separate MD simulations for both cases. Distribution curves were obtained via Gaussian broadening with default standard deviation and normalized per amount of borazine molecules, using a kernel density estimation to produce this plot.

**S5.2. Transfer of Langmuir films onto substrates**

Langmuir films were transferred onto silicon and quartz substrates via the Langmuir-Blodgett method. Si/SiO_2_ wafers were purchased from Siegert Wafer (part-no. Z14102). Quartz slides were purchased from Alfa Aesar (prod. no. 42297). These substrates were cleaned by sonicating in demi water, acetone, ethanol, and isopropanol for 10 minutes sequentially, then rinsed with isopropanol and dried with pressurized nitrogen. Before deposition, the substrates were treated with oxygen plasma for 3 min and used within a day. For uniform thin films on Si/SiO_2_, a slight contrast was visible on the optical microscope (Figure S20). The Langmuir films were transferred by the Langmuir-Schaefer technique at a constant deposition speed of 0.5 mm min^-1^ unless specified otherwise, and dried over air. Langmuir films were transferred onto copper/QUANTIFOIL TEM grids via the Langmuir-Schaefer method. R0.6/1 QUANTIFOIL on copper 200 mesh TEM grids were purchased from Van Loenen instruments (S180-1) and used without further manipulation. Glow discharging the grids was found to negatively impact the success rate of freestanding film formation. Langmuir films were transferred by the Langmuir-Schaefer technique and dried over air.


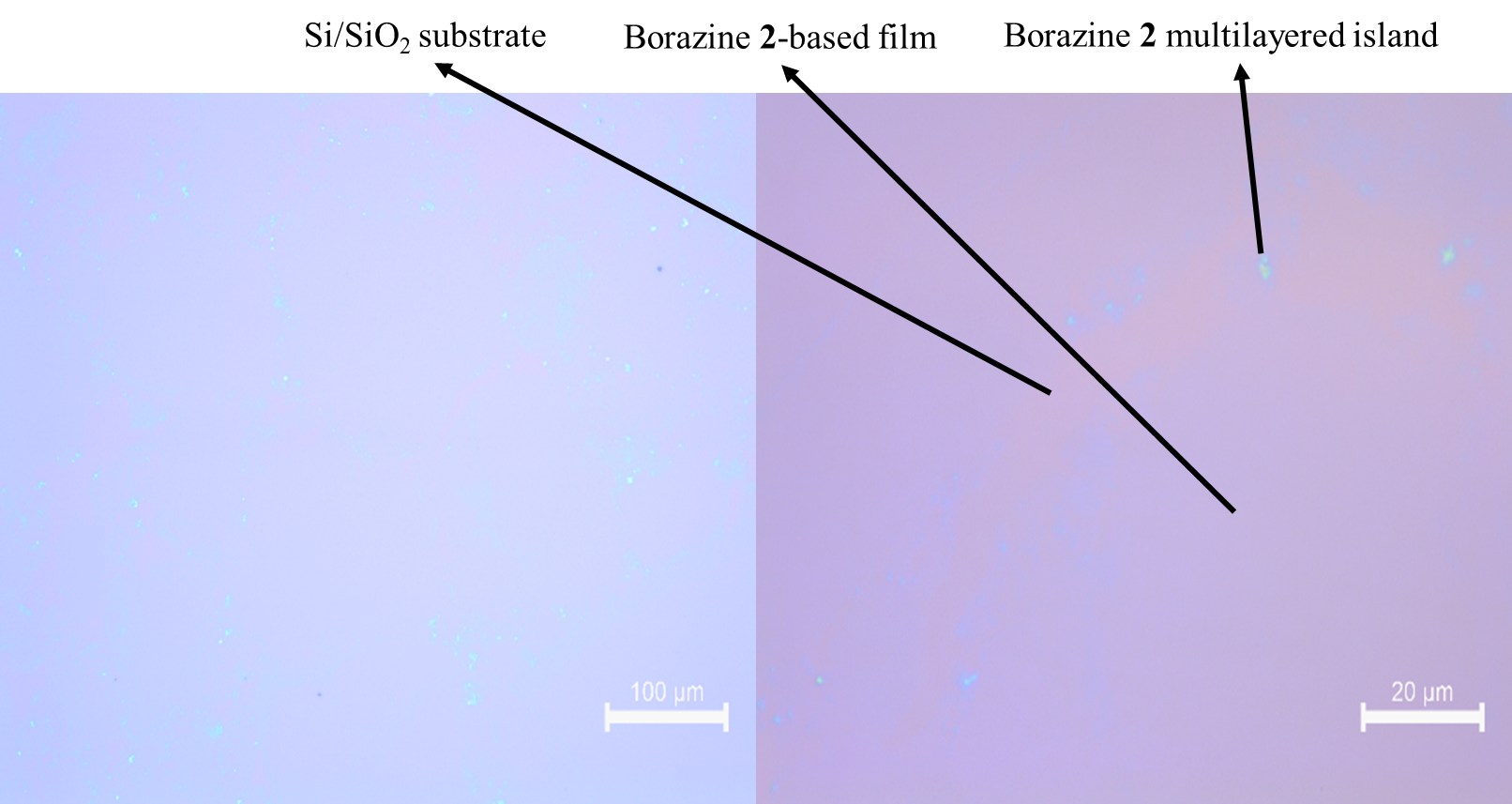


**Figure S20**. Optical images of the Langmuir-Blodgett film at **a**, 20×, and **b**, 100× magnifications. Due to the low contrast between substrate and film, we added black arrows indicating the borazine **2**-based film, the borazine **2** multilayered island, and the Si/SiO_2_ substrate.

**S6. Langmuir-Blodgett Film AFM Thickness Analysis and Freestanding Ability via SEM**

**S6.1. Thin film characterization and stability**

SEM images were measured on an Apreo SEM instrument. Typical conditions were a T2 detector with the Optiplan Use case, an acceleration voltage of 2 kV, and a beam current of 13-100 pA. Images were captured at a resolution of 1536 × 1060, dwell times of 0.2 µs, 10 integrations, and relatively high contrast and brightness levels. At lower contrast and brightness levels, the films were not visible. TEM experiments were conducted on an image-side Cs-corrected FEI Titan 80-300 microscope operated at 300 kV. AFM was measured on a JPK Nanowizard 4 Ultra Speed AFM instrument in tapping mode. Samples on Si/SiO_2_ were measured using a 160AC-NA probe from OPUS with a resonant frequency of 300 kHz and a spring constant of 26 mN m^-1^. Samples on Cu/QUANTIFOIL TEM grids were measured using an OMCL-AC240TS probe from OLYMPUS with a resonant frequency of 70 kHz and a spring constant of 2 mN m^-1^. Typically, the images were captured at a resolution of 1024 × 1024 with a line scan rate of 1 Hz. AFM images were further processed using JPK Data Processing Software. UV-Vis spectroscopy was performed on a Cary 60 (Agilent) instrument. Fluorescence spectroscopy was performed on an FLS900 fluorescence spectrometer equipped with a 450 W Xenon lamp. Solutions were measured in a quartz cuvette cell with a path length of 1 cm. Langmuir-Blodgett film and spin-coated layer were measured on a quartz slide.

Langmuir-Schaefer films were found to be unstable upon measurement with the SEM (Figure S21 b). No crystallinity could be detected from selective area electron diffraction (SAED) with the TEM (Figure S21 c). Moreover, freestanding films could not be obtained when transferring onto QUANTIFOIL containing 2 µm holes.


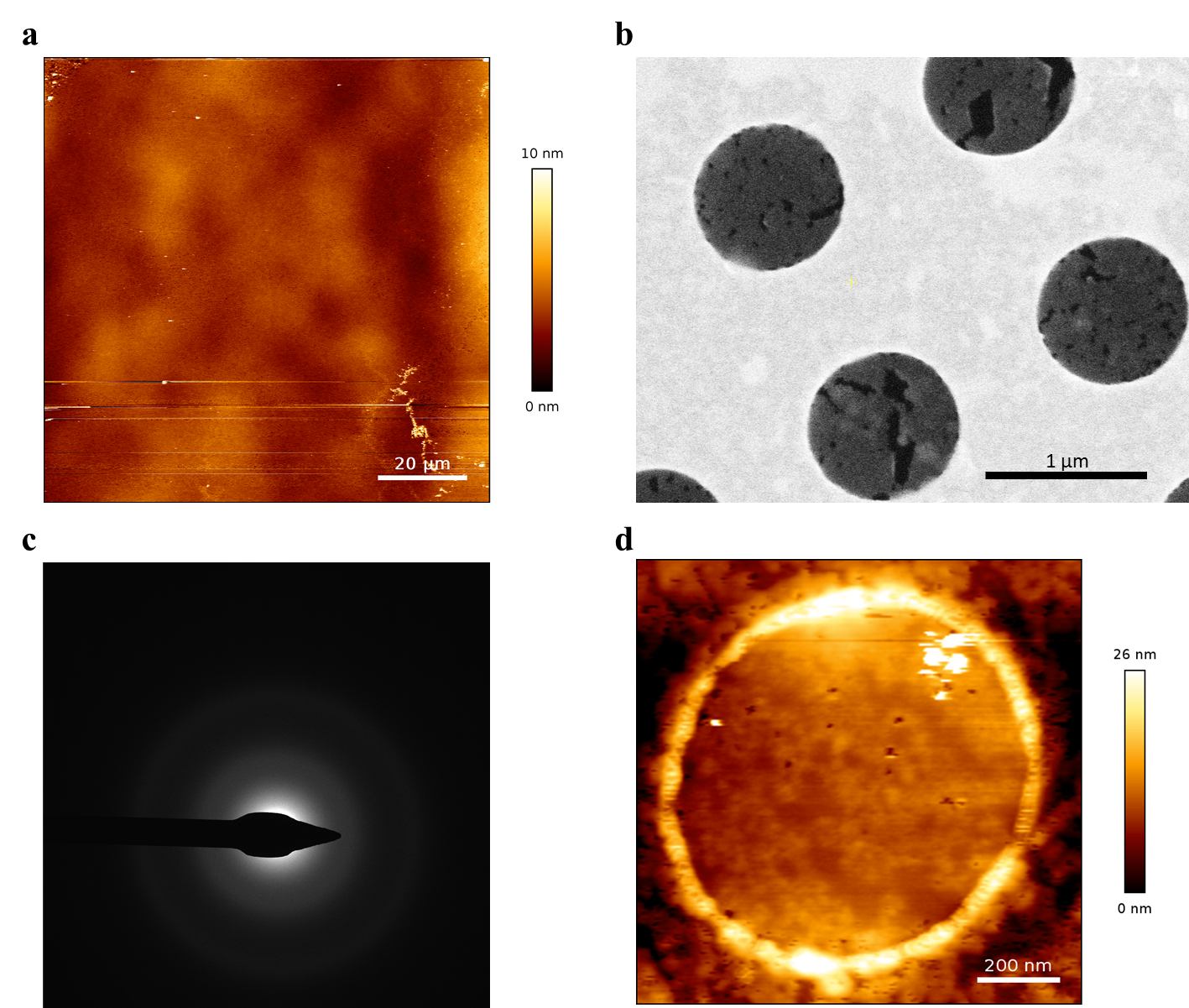


**Figure S21. a**, AFM images of a borazine Langmuir-Blodgett film prepared from 0.05 mg mL^−1^ in CHCl_3_ transferred on Si/SiO_2_ at a constant surface pressure of 2 mN m^-1^: uniform flake spanning an area of 100 × 100 µm. **b**, SEM images at 50000× magnification (blue scale bar, 1 μm) of Langmuir-Schaefer films prepared from 0.05 mg mL^-1^, compressed to 2 mN m^‑1^, on QUANTIFOIL grid perforated with an array of 0.6 µm diameter holes on a copper TEM grid. The films covering the holes were found to collapse upon prolonged electron beam irradiation. **c**, No crystallinity could be detected from SAED. **d**, High magnification of AFM analysis of Figure 5 of Langmuir-Schaefer films obtained from 0.05 mg mL^‑1^, compressed to 2 mN m^‑1^, on QUANTIFOIL containing 0.6 µm holes on a copper TEM grid before the SEM.


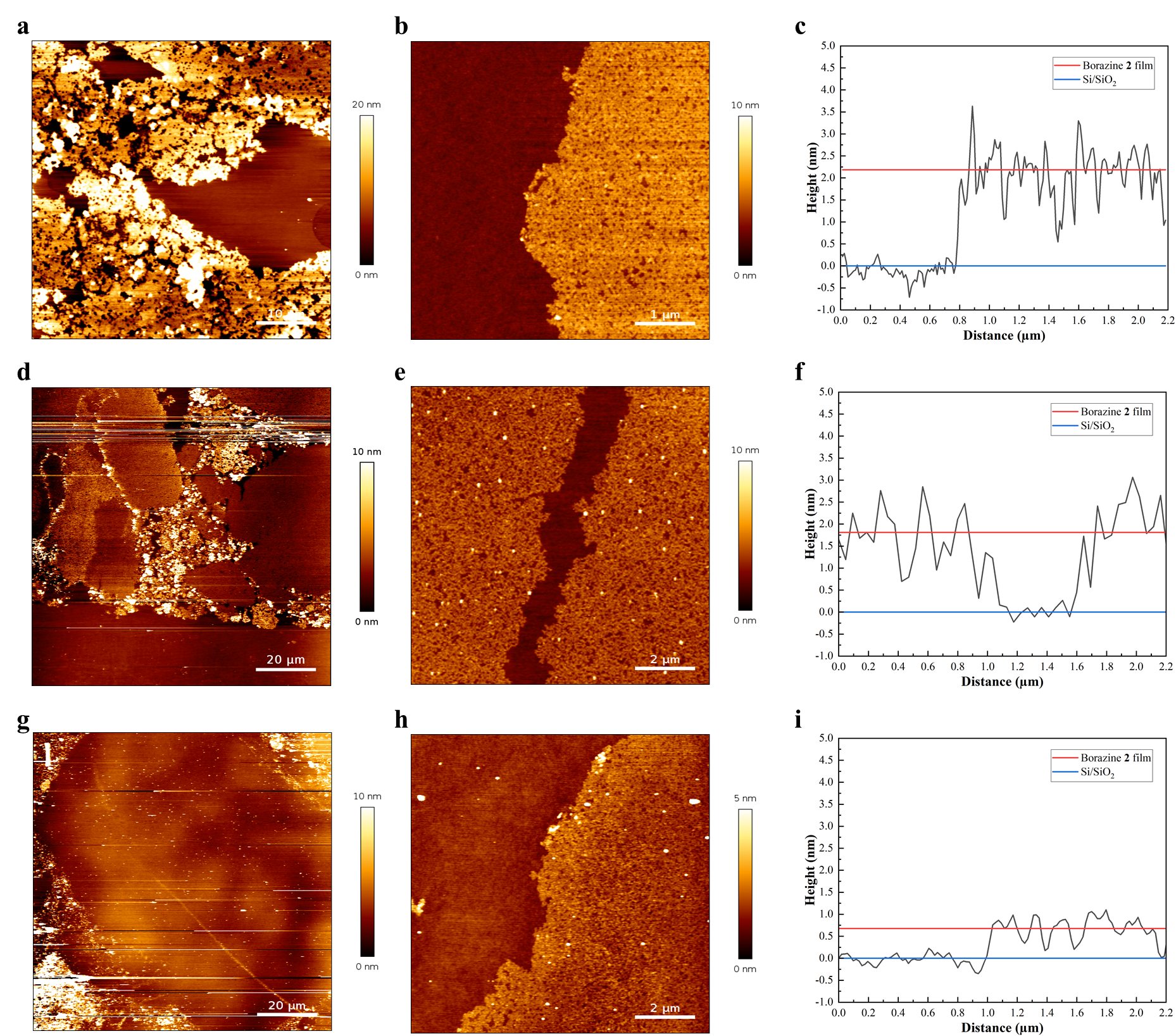


**Figure S22**. AFM image of borazine **2** films prepared from 0.05 mg mL^−1^ in CHCl_3_ transferred on Si/SiO_2_ at a constant surface pressure of 15 mN m^-1^: **a**, high degree of aggregation was found in these films between some uniform flakes at a magnification to 50 × 50 µm; **b**, a magnification to 5 × 5 µm on a crack in the film **c**, where the cross-section was plotted resulting in an estimated height of 2.2 nm. AFM image of borazine films prepared from 0.2 mg mL^−1^ in CHCl_3_ transferred onto Si/SiO_2_ at a constant surface pressure of 2 mN m^-1^: **d**, high frequency of large particles and clusters was found in these films at a magnification to 100 × 100 µm; **e**, a magnification to 10 × 10 µm on a crack in the film **f**, where the cross-section was plotted resulting in an estimated height of 1.8 nm. AFM image of borazine films prepared from 0.2 mg mL^−1^ in CHCl_3_ transferred at a constant surface pressure of 2 mN m^-1^ and fast transport rate onto the Si/SiO_2_ substrate of 2.5 mm min^-1^ compared to 0.5 mm min^-1^ of the main sample in Figure 5: **g**, high frequency of large particles and clusters was found in these films at a magnification to 100 × 100 µm; **h**, a magnification to 10 × 10 µm on a crack in the film **i**, where the cross-section was plotted resulting in an estimated height of 0.7 nm corresponding to a borazine **2**-based monolayer film.

**S7. Fluorescence Spectroscopy and TD-DFT Calculations**

To shed light on the electronic structure and optical properties of borazine **2**, time-dependent density functional theory (TD-DFT) calculations in PCM solvation were carried out (see Computational Methods in the main text). Starting from the harmonic vibrational spectra of the ground and excited states, the vibrationally resolved spectra were computed using the FCclasses3 program,^2^ and compared with experimental values. The agreement between the computed and experimental absorption and fluorescence spectra is excellent for the borazine **2** monomer (Figure 6, and Figure S23). The computational fluorescence spectra show the characteristic features of the anthracene functionalization in borazine **2** with major peaks in the range between 420-460 nm, confirming the experimental values.^3^ These results align with the optoelectronic characteristics observed in similar anthracene-based aggregates, hinting at intriguing potential applications in (opto)electronic devices.^3–5^


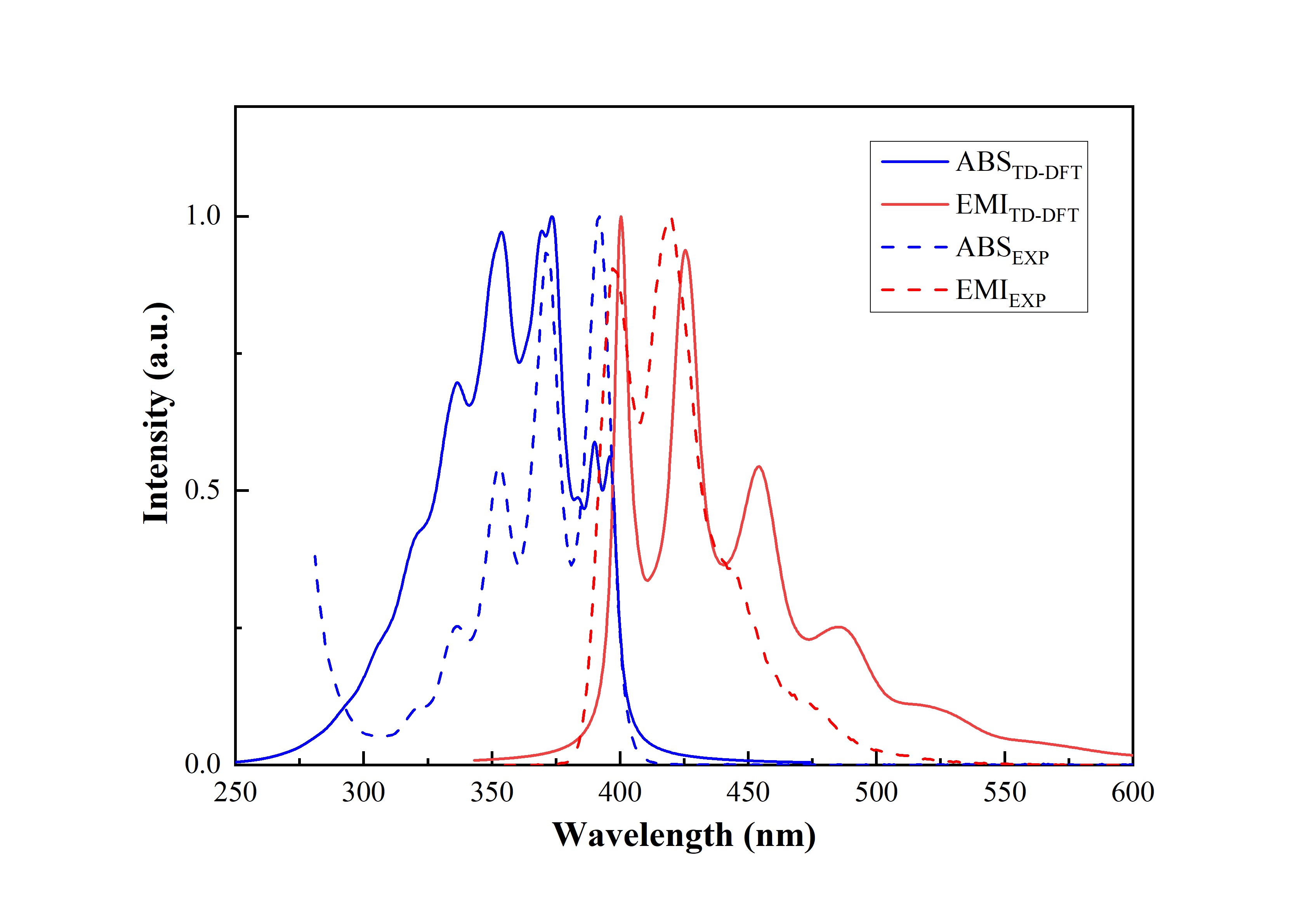


**Figure S23**. Computational (solid line) and experimental (dashed line) absorption (ABS in blue) and fluorescence (EMI in red) spectra, with intensity in arbitrary units (a.u.) and wavelength in nm. The computational spectra are calculated using the FCclasses3 program on the TD-DFT results with CAM-B3LYP functional and with Polarizable Continuum Model (PCM) CHCl_3_ solvation.^2^ The intensities of the computational absorption and fluorescence spectra are arbitrarily scaled to a maximum of 1 to facilitate the comparison with the experimental normalized spectra (Figure 6) from borazine **2** in solution (50 μM in CHCl_3_).

**References**

(1) Sheldrick, G. M. Crystal Structure Refinement with *SHELXL*. *Acta Crystallogr. C Struct. Chem.* **2015**, *71* (1), 3–8. https://doi.org/10.1107/S2053229614024218.

(2) Cerezo, J.; Santoro, F. *FCclasses3* : Vibrationally‐resolved Spectra Simulated at the Edge of the Harmonic Approximation. *J. Comput. Chem.* **2023**, *44* (4), 626–643. https://doi.org/10.1002/jcc.27027.

(3) Wakamiya, A.; Ide, T.; Yamaguchi, S. Toward π-Conjugated Molecule Bundles: Synthesis of a Series of B,B′,B″-Trianthryl-N,N′,N″-Triarylborazines and the Bundle Effects on Their Properties. *J. Am. Chem. Soc.* **2005**, *127* (42), 14859–14866. https://doi.org/10.1021/ja0537171.

(4) Kervyn, S.; Fenwick, O.; Di Stasio, F.; Shin, Y. S.; Wouters, J.; Accorsi, G.; Osella, S.; Beljonne, D.; Cacialli, F.; Bonifazi, D. Polymorphism, Fluorescence, and Optoelectronic Properties of a Borazine Derivative. *Chem. Eur. J.* **2013**, *19* (24), 7771–7779. https://doi.org/10.1002/chem.201204598.

(5) Servalli, M.; Celebi, K.; Payamyar, P.; Zheng, L.; Položij, M.; Lowe, B.; Kuc, A.; Schwarz, T.; Thorwarth, K.; Borgschulte, A.; Heine, T.; Zenobi, R.; Schlüter, A. D. Photochemical Creation of Covalent Organic 2D Monolayer Objects in Defined Shapes *via* a Lithographic 2D Polymerization. *ACS Nano* **2018**, *12* (11), 11294–11306. https://doi.org/10.1021/acsnano.8b05964.
